# Genetic diversity and population structure of *Tenacibaculum maritimum*, a serious bacterial pathogen of marine fish: from genome comparisons to high throughput MALDI-TOF typing

**DOI:** 10.1186/s13567-020-00782-0

**Published:** 2020-05-07

**Authors:** Sébastien Bridel, Frédéric Bourgeon, Arnaud Marie, Denis Saulnier, Sophie Pasek, Pierre Nicolas, Jean-François Bernardet, Eric Duchaud

**Affiliations:** 1 Université Paris-Saclay, INRAE, UVSQ, VIM 78350 Jouy-En-Josas, France; 2 Labofarm, Finalab, 22603 Loudéac, France; 3 Université de Versailles Saint-Quentin-En-Yvelines, 78180 Montigny-Le-Bretonneux, France; 4 Bio Chêne Vert, Finalab, Rue Blaise Pascal, 35220 Châteaubourg, France; 5 Ifremer, UMR EIO 241, Labex Corail, Centre du Pacifique, BP 49, Taravao, 98719 Tahiti, French Polynesia; 6 Institut de Systématique Evolution, Biodiversité, UMR 7205 Sorbonne Université MNHN CNRS EPHE, Paris, France; 7 Université Paris-Saclay, INRAE, MaIAGE 78350 Jouy-en-Josas, France

## Abstract

*Tenacibaculum maritimum* is responsible for tenacibaculosis, a devastating marine fish disease. This filamentous bacterium displays a very broad host range and a worldwide geographical distribution. We analyzed and compared the genomes of 25 *T. maritimum* strains, including 22 newly draft-sequenced genomes from isolates selected based on available MLST data, geographical origin and host fish. The genome size (~3.356 Mb in average) of all strains is very similar. The core genome is composed of 2116 protein-coding genes accounting for ~75% of the genes in each genome. These conserved regions harbor a moderate level of nucleotide diversity (~0.0071 bp^−1^) whose analysis reveals an important contribution of recombination (r/m ≥ 7) in the evolutionary process of this cohesive species that appears subdivided into several subgroups. Association trends between these subgroups and specific geographical origin or ecological niche remains to be clarified. We also evaluated the potential of MALDI-TOF-MS to assess the variability between *T. maritimum* isolates. Using genome sequence data, several detected mass peaks were assigned to ribosomal proteins. Additionally, variations corresponding to single or multiple amino acid changes in several ribosomal proteins explaining the detected mass shifts were identified. By combining nine polymorphic biomarker ions, we identified combinations referred to as MALDI-Types (MTs). By investigating 131 bacterial isolates retrieved from a variety of isolation sources, we identified twenty MALDI-Types as well as four MALDI-Groups (MGs). We propose this MALDI-TOF-MS Multi Peak Shift Typing scheme as a cheap, fast and an accurate method for screening *T. maritimum* isolates for large-scale epidemiological surveys.

## Introduction

The rapid development of intensive aquaculture has been associated with a dramatic increase in outbreaks of infectious diseases [1, 2]. Additionally, the international spread of pathogens through the trade of fish and eggs or as a response to environmental changes has been documented for some important viruses and bacteria [3, 4]. In this context, the success and sustainability of aquaculture largely depend on the understanding of the evolution and epidemiology of pathogens [1]. Among those, several species of the genus *Tenacibaculum* (family *Flavobacteriaceae*, phylum *Bacteroidetes*) are responsible for diseases collectively designated as tenacibaculosis, a very serious bacterial condition of many commercial marine fish species leading to considerable economic losses [5, 6]. *Tenacibaculum maritimum* (formerly *Flexibacter maritimus*) was the first species to be characterized and probably the best-known pathogen in the genus. Moreover, *T. maritimum* can affect many feral, captive, and cultured fish species [5, 7] and has been repeatedly identified in many marine aquaculture systems worldwide. Diseased fish usually exhibit a diversity of external symptoms including corroded mouth, skin ulcers, fin necrosis, and rotted tail. Skin lesions are often colonized by opportunistic pathogens such as *Vibrio* spp. So far, only one vaccine is commercially available, but it is restricted to the protection of turbot. Hence, the control of *T. maritimum* outbreaks essentially relies on the use of antibiotics, sometimes combined with external disinfectants [8].

Reliable methods for studying the relationships between isolates of the same bacterial species (i.e., strain typing) are a key step for understanding the population structure, the spreading and the epidemiology of pathogens. Different typing methods have been proposed for epidemiological investigations of *T. maritimum* [9]. Three serotypes displaying varying degrees of association with host fish species have been reported [10, 11] and different molecular technics have been used to determine the intraspecific diversity of *T. maritimum* [12, 13]. Serological data were compared with several PCR-based methods [14]. In 2014, we proposed a Multi Locus Sequence Analysis (MLSA) scheme [15] that proved to be a powerful discriminating tool for isolate identification and taxonomic affiliation [16, 17]. MLSA revealed an unforeseen diversity including several, yet undescribed, pathogenic species in Norway [18]. In addition, this 11-locus sequenced-based method revealed a high number of distinct genotypes for the species *T. maritimum*, suggesting an endemic distribution of strains without significant contribution of long-distance dissemination linked to international fish movements. More recently, whole-cell matrix-assisted laser desorption ionization–time of flight mass spectrometry (MALDI-TOF MS) was used for the differentiation of several fish-pathogenic *Tenacibaculum* species [14, 19]. However, these studies did not reveal any relationships between the proteomic profiles and the source of isolation of the strains for any of the *Tenacibaculum* species analyzed, and no biomarker below the species level (e.g., serotype-specific peaks) was detected for *T. maritimum*. Meanwhile, the complete genome of the *T. maritimum* type strain [20] as well as the draft genomes of the type strains and several field isolates of *T. dicentrarchi* and “*T. finnmarkense*” have been recently published [21], paving the way to comparative genomics.

In this study, we analyzed and compared 25 genomes (including 22 newly draft-sequenced) of *T. maritimum* isolates from various geographical origins and host fish species to draw a global picture of the genomic diversity of the species. In addition, we developed a genome-based MALDI-TOF MS scheme and typed 131 field isolates. While this technique is commonly used for species identification, bacterial characterization below the species level is much more challenging, requiring the identification of subtle differences between strains [22]. We also propose a dedicated website that allows both identification and typing of new *Tenacibaculum* isolates [23].

## Materials and methods

### Bacterial strains

*Tenacibaclum maritimum* strains were grown in marine 2216E broth (Difco, Becton, Dickinson and Co., Franklin Lakes, New Jersey, USA) for 24 h at 28 °C and 170 rpm. Stock cultures were preserved in marine 2216E broth containing 20% (v/v) glycerol at −80 °C. The 25 strains used in this study are listed in Table 1 and the bacterial isolates subjected to MALDI-TOF MS analysis are listed in Additional file 1.

**Table 1:** **General genome features.**

Strain	Country	Host	Tissue	Date of isolation	Technology	Reads (post-trimming)	Contigs (> 2000pb)	Total length	GC %	Coverage	Number of predicted genomic islands	SNPs vs. NCIMB 2154^T^	Predicted CDS	Genbank Assembly
NCIMB 2154T	Japan	*Pagrus major*	Skin	1977	[20]	n/a	1	3453 971	32.01	1734	29	0	2774	GCA_900119795.1
TM-KORJJ	Korea	*Paralichthys olivaceus*	n/a	n/a	PacBio	n/a	1	3333 272	31.98	300	24	15 049	2735	GCA_004803875.1
NBRC 15946	Japan	n/a	n/a	n/a	HiSeq	n/a	96	3 240 791	31.80	123	20	10 084	2692	GCA_000509405.1
P1-39	France	*Dicentrarchus labrax*	Liver	2010	HiSeq (2 × 100 bp)	56 562 140	104	3 337 690	31.79	93	23	15 401	2788	GCA_902705535
P4-45	France	*Dicentrarchus labrax*	Skin	2010	HiSeq (2 × 100 bp)	54 681 392	73	3 349 546	31.81	70	27	15 707	2843	GCA_902705495
902	France	*Dicentrarchus labrax*	Skin	2013	HiSeq (2 × 100 bp)	88 844 854	68	3 372 337	31.80	85	24	15 521	2851	GCA_902705365
Aq16-85	French Polynesia	*Platax orbicularis*	Skin	2016	MiSeq (2 × 300 bp)	2 174 369	43	3 198 696	31.88	220	21	14 929	2664	GCA_902705305
Aq16-88	French Polynesia	*Platax orbicularis*	Skin	2016	MiSeq (2 × 300 bp)	3 434 486	45	3 196 671	31.88	301	21	14 966	2665	GCA_902705275
Aq16-89	French Polynesia	*Platax orbicularis*	Skin	2016	MiSeq (2 × 300 bp)	2 156 781	45	3 196 642	31.89	171	21	14 914	2666	GCA_902705375
TFA4	French Polynesia	*Platax orbicularis*	Skin	2013	MiSeq (2 × 300 bp)	2 369 550	66	3 356 632	31.82	226	33	14 961	2865	GCA_902705565
FS08(1)	Italy	*Sparus aurata*	Skin	2006	MiSeq (2 × 300 bp)	1 256 466	54	3 399 437	31.81	87	30	4424	2866	GCA_902705395
NAC SLCC MFF	Malta	*Dicentrarchus labrax*	Skin	1995	MiSeq (2 × 300 bp)	1 091 150	80	3 352 671	31.81	76	29	40 598	2795	GCA_902705345
USC SP9.1	Spain	*Salmo salar*	Skin	1993	MiSeq (2 × 300 bp)	727 120	80	3 395 385	31.84	46	30	31 252	2779	GCA_902705515
DPIF 89/3001-6.2	Tasmania	*Latris lineata*	Skin	1989	MiSeq ( 2 × 300 bp)	1 019 320	129	3 448 890	31.77	66	33	26 962	2788	GCA_902705315
DPIF 89/0239-1	Tasmania	*Salmo salar*	Skin	1989	MiSeq (2 × 300 bp)	1 496 188	55	3 353 931	31.90	92	28	17 743	2773	GCA_902705355
USC SE30.1	Spain	*Oncorhynchus kisutch*	Mouth	1993	MiSeq (2 × 300 bp)	749 406	111	3 544 405	31.75	52	30	31 928	2928	GCA_902705525
UCD SB2	California	*Atractoscion nobilis*	n/a	1995	MiSeq (2 × 300 bp)	1 323 140	50	3 308 376	31.91	85	23	17 064	2715	GCA_902705445
JIP 32/91-4	France	*Dicentrarchus labrax*	Skin	1991	MiSeq (2 × 300 bp)	1 031 822	59	3 447 003	31.80	59	31	17 265	2872	GCA_902705385
CVI1001048	Holland	*Solea solea*	Skin	2010	MiSeq (2 × 300 bp)	1 242 180	42	3224 047	31.94	90	16	1659	2670	GCA_902705265
FC	Chile	*Scophthalmus maximus*	Eye	1998	MiSeq (2 × 300 bp)	1086 816	57	3 505 634	32.01	70	27	16 571	2921	GCA_902705415
P2-48	France	*Solea senegalensis*	Skin	2010	MiSeq (2 × 300 bp)	1 349 672	55	3 418 994	31.86	90	34	43 607	2910	GCA_902705555
P2-27	Spain	*Scophthalmus maximus*	Skin	2011	MiSeq (2 × 300 bp)	1 087 036	88	3 371 677	31.82	76	24	19 639	2821	GCA_902705465
JIP 46/00	France	*Scophthalmus maximus*	Skin	2000	MiSeq (2 × 300 bp)	1 155 334	56	3 371 335	31.89	73	25	17 215	2781	GCA_902705435
JIP 10/97	France	*Scophthalmus maximus*	Skin	1997	MiSeq (2 × 300 bp)	1 345 346	52	3 333 073	31.86	86	22	17 441	2746	GCA_902705285
NCIMB 2158	Scotland	*Solea solea*	Skin	1981	MiSeq (2 × 300 bp)	1 138 826	74	3 369 590	31.87	79	28	17 387	2797	GCA_902705425

The list of contributors is available in Additional file 1.

### Genome sequencing, assembly and annotation

Following centrifugation of the liquid culture, genomic DNA was extracted from the pellet using the Wizard genomic DNA purification kit (Promega, Madison, Wisconsin, USA). The genomes were sequenced using Illumina (HiSeq, 100 paired-end or MiSeq, 300 paired-end) and genome assemblies were performed using Spades and Velvet on the PATRIC website with default settings [24]. The resulting contigs (> 2000 bp) were integrated into the MicroScope platform [25].

### Genome analysis and comparisons

Average Nucleotide Identity analyses were performed using the ANIm method [26] with the Python module Pyani [27] using proposed threshold of ≈ 95–96% for species delineation. Genome annotation, including manual curation, and comparisons, including pan and core genome computation, were performed using the web interface MicroScope [28], which allows graphic visualization enhanced by a synchronized representation of synteny groups [29]. Persistent, shell and cloud genomes were computed using the PPanGGOLiN 0.1.4 software [30]. Considering the low levels of sequence divergence at typical core genome loci previously reported for *T. maritimum* [15], we chose a cutoff of 80% identity and 80% on the minimal coverage of the length between the aligned portions of two proteins to determine whether two CDSs were members of the same gene family.

The 25 genomes were aligned using Snippy [31] with the genome of strain NCIMB 2154^T^ (the type strain of *T. maritimum*) [20] serving as a reference. The resulting whole-genome alignment was used for phylogenetic tree reconstruction using Gubbins [32] and the Phylip package version 3.6 released by Felsenstein in 2005, available online at [33] and originally released in 1980 [34]. Regions of high diversity (high number of single nucleotide polymorphisms [SNPs]) presumably linked to recombination events were masked at each Gubbins iteration. A Maximum Likelihood tree was built at the fifth iteration using non-masked polymorphic sites. Neighbor-joining and parsimony trees were obtained with Phylip suite v3.696 (programs dnadist, neighbor, and dnapars) after removing positions with gaps in the alignment. Custom Perl and R scripts were used for the nucleotide diversity analysis (computation of the average pairwise nucleotide diversity and of the homoplasy index) and graphical representations (including R library “ape” for the drawing of phylogenetic trees). An estimate of the ratio of recombination and mutation (r/m) based on the analysis of SNPs between pairs of closely related isolates was obtained using a two-state hidden-Markov model (HMM), as described in Duchaud et al. [35].

### Preparation of bacterial samples for MALDI-TOF MS

The ethanol/formic acid extraction procedure, as described by Mellmann et al. [36] was used. Briefly, a colony of a fresh overnight culture was inoculated in 5 mL marine 2216E broth and cultivated at 28 °C for 24 h with shaking (170 rpm). The resulting bacterial culture was centrifuged at 12 000 *g* in a desktop centrifuge for 2 min and the supernatant discarded. About 10 mg of the resulting bacterial pellet was transferred in a clean Eppendorf tube with 300 μL of ultra-pure water (Acros organics, New Jersey, USA) and vigorously mixed to resuspend the cells. 900 μL of 100% ethanol (VWR Chemicals, Radnor, Pennsylvanie, USA) was added into the tube and mixed again. The mix was directly processed or kept at room temperature (RT) up to 1 month for the assessment of the ethanol-fixed bacteria protocol. The tube was centrifuged at 12 000 *g* in a desktop centrifuge for 2 min and the supernatant discarded. The tube was centrifuged for 2 additional minutes and the residual ethanol removed and the pellet was dried at RT. Thirty μL of 70% formic acid was added and mixed thoroughly by pipetting. An equal volume of acetonitrile was then added to the tube, mixed carefully and then centrifuged at 12 000 *g* in a desktop centrifuge for 2 min. One μL of the supernatant was dropped onto a 96-spot polished steel target. The sample spot was dried at RT and 1 μL of matrix solution (α-cyano-4-hydroxycinnamic acid in 50% acetonitrile, 47.5% water and 2.5% trifluoroacetic acid) was then added. The sample spot was finally air dried again before analysis. A calibration of the MALDI-TOF mass spectrometer was performed using Bruker bacterial test standard for each series of acquisitions with a mass tolerance limit of ± 300 parts-per-million (ppm). To evaluate alternative sample preparation procedures, bacteria were cultivated on solid medium [i.e., marine 2216E broth and 15 g/L agar (Invitrogen, Illkirch, France)] at 28 °C for 24 h. About 10 bacterial colonies were collected and processed as the bacterial pellet obtained from liquid culture. For the assessment of the direct colony picking protocol, a single bacterial colony was picked with a sterile toothpick and directly deposited onto a 96-spot polished steel target and processed as the sample spot previously mentioned.

### MALDI-TOF MS data acquisition

A MALDI Biotyper Microflex LT controlled by Compass flexControl software (version 3.4; Bruker Daltonics, Billerica, Massachusetts, USA) was used to generate mass spectra for all isolates. Mass spectra were acquired using automatic mode and default settings (2000 to 20 0000 Da; linear positive mode; 240 laser shots). For each isolate, twelve spectra (four spots of each isolate extracted were measured 3 times) were recorded.

### Peak shift characterization using genomic data

Ribosomal protein sequences were retrieved from the MicroScope annotation platform using the 25 available *T. maritimum* genomes. The theoretical mass weight for each sequence was computed with the *mw*() function from the Peptides R-package and TermiNator [37] was used to predict the first methionine cleavage resulting in a theoretical loss of 131.2 Da. The theoretical masses obtained were compared to the peak list and spectra were screened to retrieve peak shifts using the predicted masses of presumptive polymorphic biomarkers. Several other peak shifts were identified during this step but were not kept for typing purpose because of the stringent criteria used (see section “Results”).

### MALDI-TOF data analysis algorithm and implementation in R

We used different R packages dedicated to mass spectrometry analysis from the BioConductor repository [38], i.e. MALDIquant Foreign, MALDIquant, MALDIrppa and MassSpecWavelet. Using these packages, any type of MALDI-TOF data can be loaded from any manufacturer. Raw data generated by Microflex® Bruker were imported into R environment by MALDIquant Foreign. The spectra pre-processing steps (i.e., intensity transformation, baseline correction, intensity correction, spectra alignment, peak detection), curve smoothing and peak detection on average spectra (mean spectra) were performed using MALDIquant, MALDIrppa and MassSpecWavelet [39], respectively. This method is based on local maxima detection at different scales, and the retained peaks are observed at many scales as true maxima (true peaks). Thus, it theoretically gets rid of noisy peaks more efficiently than classical algorithms (e.g., about 300 peaks were detected with MALDIquant/MALDIrppa with soft parameters whereas around 100 peaks were detected with MassSpecWavelet for each *T. maritimum* average spectrum). The resulting peak list is then compared to a reference peak list that encompasses either: (i) full spectra of the type strains of *Tenacibaculum* species, (ii) species-specific biomarkers or (iii) subtyping biomarkers. We considered two peaks as matching between a sample and the reference with a tolerance of 700 ppm. MALDIquantTypeR is a home-made R application developed for MALDI type assignment [23].

## Results

### General genome features

The origins of the sequenced genomes, the main sequencing and assembly data and the genomic characteristics are listed in Table 1. The number of contigs (> 2 kb) for the 22 newly sequenced genomes varied from 42 to 129 depending on sequencing technology, sequencing depth and genome properties (i.e., number of repeats). Genome length estimated by the cumulated size of the contigs was 3356 Kb in average with very little variations between isolates (standard deviation (SD) = 92 Kb). Each genome contained an average of 2788 (SD = 81) predicted CDSs. The core-genome was composed of 2116 gene families (out of 5809 gene families in total), representing about 75% of the CDSs in each genome. The core-genome encompassed all the predicted toxins and virulence factors (i.e., cholesterol-dependent cytolysin, collagenase, sphingomyelinase, ceramidase, chondroitin AC lyase, streptopain family protease, sialidase, iron uptake systems and T9SS components) previously identified in strain NCIMB 2154^T^ [20]. In addition, the persistent-genome, equivalent to a relaxed core-genome (i.e., genes conserved in all but a few genomes) encompassed about 2500 gene families representing about 81% of the CDSs. There were about 10% of shell-genome genes (i.e., genes having intermediate frequencies corresponding to moderately conserved genes potentially associated to environmental adaptation capabilities) and about 9% of cloud-genome (i.e., genes found at a very low frequency). These two later values were likely over-estimated since the newly sequenced genomes were not fully assembled and because of pseudogenization events and gaps between contigs that lead to gene fragments that artificially increase the total number of shell-genome and cloud-genome gene families. Strikingly, most of the shell- and cloud-genome genes were encompassed in genomic islands (region of genomic plasticity). Each genome contained an average of 26 (SD = 4.65) genomic islands (Table 1). Interestingly, strain FC isolated in Chile encompassed a unique (i.e., not identified in other strains), 83-kb long (MARITFC_v1_10015 to MARITFC_v1_10098) genomic island that displayed prophage characteristics (i.e., located at an Arg tRNA and bordered with an integrase/recombinase encoding gene). This island contained many genes predicted to be involved in heavy metal resistance, including genes encoding proteins of the copper oxidase family and different cobalt-zinc-cadmium efflux pumps.

We focused on the two fully PacBio-assembled genomes (i.e., strains NCIMB 2154^T^ and TM-KORJJ) contained in our dataset to accurately identify genes encompassed in these islands. Strain NCIMB 2154^T^ contained 29 genomic islands (encompassing 384 genes and corresponding to 13% of the total number of CDSs) while strain TM-KORJJ contained 24 genomic islands (encompassing 351 genes and corresponding to 12% of the total number of CDSs). Most of these islands displayed classical prophage characteristics and encompassed CDSs encoding for phage structural proteins, integrases, transposases, insertion sequences, restriction/modification systems and Vgr/Rhs elements or their scars.

### Nucleotide diversity and population structure

To estimate the nucleotide diversity, we first used assembled draft genomes and computed the average nucleotide identity (ANI) between pairs of genomes. ANI values ranged between 98.18% and 100% (Additional file 2), far above the species delineation threshold of 95–96% [26], revealing a cohesive species and confirming that all the isolates included in this genomic study indeed belonged to the species *T. maritimum*.

Using a whole genome alignment built on strain NCIMB 2154^T^ as a reference and corresponding to 2587 083 bp of conserved concatenated sequence, we identified a total of 86 217 SNPs (mean 21 126 SNPs ± SD 9795) out of which 84 694 (98.2%) where bi-allelic. The average pairwise nucleotide divergence between isolates (Table 1) amounted to 18 496 SNPs corresponding to a diversity π of 0.0071 bp^−1^ with a maximum of 0.0152 bp^−1^. The smallest divergence was 0.0021 bp^−1^ (5477 SNPs), reflecting an absence of very closely related isolate pairs outside of the three isolates Aq18-85, Aq18-88 and Aq18-89 (no SNPs found between them).

**Figure 1:**
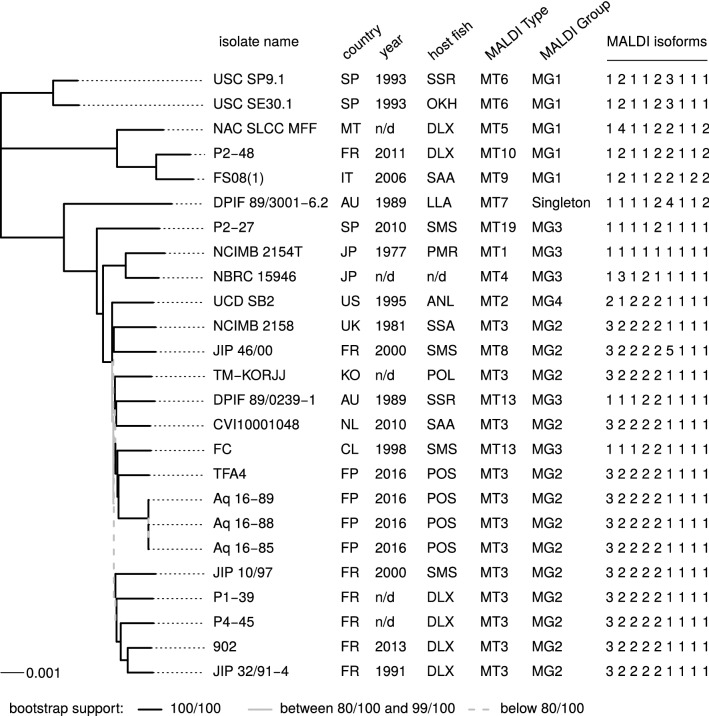
**Neighbor-joining tree based on a Jukes-Cantor distance of the 25*T. maritimum*genomes.** The tree is based on snippy alignment without gap regions. It is reconstructed by NJ method using a Jukes-Cantor distance. 100 bootstrap replicates were made. A black line denotes a branch support of 100/100, while a grey line denotes a value between 80/100 and 99/100 and a grey dotted line a value below 80/100. The origin of strains is indicated next to the tree and the meanings of each abbreviation are detailed below. The MALDI-Type, the MALDI-Group and the corresponding MALDI isomorphic profile are plotted on the right hand side of the figure. Origin: Australia (Tasmania), AU; Chile, CL; France, FR; French Polynesia, FP; Italy, IT; Japan, JP; Malta, MT; Spain, SP; United Kingdom (Scotland), UK; USA (California), US; no data available, n/d. Fish host species: ASI, *Acanthopagrus shlegeli*; ANS, *Atrasctoscion nobilis*; CTS, *Carcharias taurus*; DLX, *Dicentrarchus labrax*; EMX, *Engraulis mordax*; LLA, *Latris lineata*; OKH, *Onchorynchus kisutch*; OMS, *Onchorynchus mykiss*; PMR, *Pagrus major*; POL, *Paralichthys olivaceus*; POS, *Platax orbicularis*; SSR, *Salmo salar*; SMS, *Scophtalmus maximus*; SQA, *Seriola quinqueradiata*; SSS, *Solea senegalensis*; SAA, *Sparus aurata*; n/d no data available.

The whole genome alignment was subjected to several tree reconstruction methods. Trees obtained by parsimony or with the more sophisticated Gubbins method that attempts to remove recombination tracts are shown in Additional files 3 and 4, respectively. These trees tended to contain internal branches of substantial length with poor bootstrap values (see parsimony tree S3A) presumably due to recombination. To reflect more faithfully the pairwise distances between isolates, Figure 1 presents a neighbor-joining tree based on a simple Jukes-Cantor distance. In this tree, whose topology is very similar to those of the Parsimony and Gubbins trees, branches with poor bootstrap support tended to disappear (i.e. to have length close to zero). Overall, the topologies and bootstrap values supported the division in three subgroups (corresponding to clades A, B and C) previously observed using MLST data [15]. Clades A, B and C encompassed 3, 20 and 2 strains, respectively. The core genome-based tree and the previously obtained MLST-based tree [15] showed similar clade composition with the only exceptions of strains DPIF 89/3001-6.2 and DPIF 89/0239-1 that belong to ST24 and ST20, respectively (MLST subgroup C) whereas they belong to core genome clade B. However, the position of these two strains had very poor bootstrap support in the MLST-based tree.

Pervasive recombination was obvious from the difference between the 158 863 changes along the parsimony tree and the 87 770 changes that would have been needed in the absence of homoplasy and recombination at the 86 217 polymorphic positions (considering 1493 tri- and 30 quadri-allelic SNPs). These numbers corresponded to an apparent homoplasy index HI of 44.8% [(158 863–87 770)/158 863]. A similar HI of 44.3% [(102 755–57 185)/102 755] was obtained when examining polymorphism only within clade B (the average pairwise nucleotide divergence measured in this clade was 0.0042 bp^−1^). To further quantify the impact of recombination, we analyzed the pattern of pairwise nucleotide divergence between closely related genomes, selecting unambiguous pairs of tips in the reconstructed trees such as to be able to distinguish private and shared polymorphism. In practice, we used for this purpose the comparison USC SP9.1 vs. USC SE30.1 (5615 SNPs) and FS08(1) vs. P2−48 (7980 SNPs). No isolates from clade B satisfied our needs of forming a clearly isolated pair. Only bi-allelic SNPs were used, and the fraction of shared polymorphism (i.e. polymorphism also found outside the pair) represented as much as 78.8% of the sites that distinguished the first pair of isolates and 69.3% for the second pair, indicating a considerable contribution of recombination to divergence since mutation is expected to produce almost exclusively private polymorphism while recombination is expected to produce tracts mixing shared and private polymorphism. A hidden Markov model served to delineate recombination tracts and lead to estimates of the ratio of recombination and mutations to nucleotide-level divergence (r/m) of 20.6 for USC SP9.1 vs. USC SE30.1 and 7.7 for FS08(1) vs. P2−48 (Additional files 5A and B, respectively).

As observed on the phylogenetic trees, strains retrieved from the same geographical origin tended to cluster together, indicating genetic relatedness. Indeed, the two strains originating from the Atlantic coast of Spain (USC SE30.1 and USC SP9.1) formed cluster C while the three strains in group-A [FS08(1), NAC SLCC MFF and P2-48] were all retrieved from countries bordering the Mediterranean Sea. The two Japanese strains NCIMB 2154^T^ and NBRC 15946 also appeared related. The three strains Aq16-85, Aq16-88 and Aq16-89 isolated in French Polynesia in 2016 were virtually identical to each other (0 SNPs in the 2587 083 aligned positions of the core-genome), suggesting a dissemination of a single clone between fish farms where *Platax orbicularis* are raised. In addition, these three strains were related to strain TFA4 also isolated in French Polynesia in 2013. The Chilean strain FC, also originating from the South Pacific, belonged to the same group. Five strains (i.e., JIP 10/97, P1-39, P4-45, 902 and JIP 32/91-4) retrieved from France over 22 years were also grouped together in the NJ tree (Figure 1).

### Identification of potential biomarkers in genomic data

Because genomic data pointed to a cohesive species but still displaying variability to some extent, we aimed to evaluate the potential of MALDI-TOF MS for rapid screening and typing using specific sets of biomarker ions. Since half of the detected peaks in bacterial MALDI-TOF MS spectra correspond to ribosomal proteins [40], we focused on this protein set. Using genome sequence data, we first retrieved all deduced ribosomal protein sequences and predicted the first methionine removal using the Terminator software [37, 41]. The molecular weight of each deduced ribosomal protein was computed. We retrieved ribosomal protein sequences without polymorphism (i.e., invariant ribosomal protein sequences hereafter designed as monomorphic) that could serve as species biomarkers and for MALDI-TOF MS spectra internal calibration. We also retrieved ribosomal protein sequences displaying polymorphism (i.e., ribosomal protein sequences with variation hereafter designed as polymorphic) since they are likely relevant biomarkers for strain typing. Strikingly, one-third (18/54) of the ribosomal proteins were monomorphic while the others (36/54) displayed some degree of amino-acid polymorphism that mostly gave rise to a mass change (the resulting information is summarized in Additional file 6). Strikingly, the topology of the hierarchical classification tree deduced from this data set was globally congruent with that of the trees obtained using the core genome genes (Figure 1 and Additional file 3). Indeed, some ribosomal protein-encoding genes displayed clade-specific variations. For example, gene *rplU* encoded two different versions of the 50S ribosomal subunit protein L21: a 209 amino-acid long RplU protein in clades A and C and a 161 amino-acid long RplU protein in clade B. Other examples were provided by proteins RpmI and RpsP that displayed isoforms only found in clade A or by RplD that displayed an isoform only found in clade C.

### Selection of invariant biomarker ions for internal calibration of spectra and species identification

Using our monomorphic biomarker candidates, we performed visual inspection of spectra obtained from 24 out of the 25 genome-sequenced strains to identify the corresponding peaks. We selected 18 peaks (Additional file 7) according to the following stringent criteria: (i) they covered as much of the entire spectra as possible (ranging from 2958 m/z to 12 460 m/z); (ii) they corresponded to mono-, di- or tri-charged monomorphic ribosomal proteins; (iii) they occurred in all MALDI-TOF spectra of the 24 isolates; (iv) most of them were of high intensity (ranging from 98 to 1371, mean intensity = 547), even at both ends of the spectra (though intensity was globally lower at these m/z locations); and (v) they were not disrupted by the presence of other very close peaks which could degrade peak detection reliability; in other words, retained peaks had to be in a window devoid of additional peaks. Only two selected peaks (i.e., RpmJ-M-H1 and RpmE-M-H2) represented exceptions to the latter criterion. Indeed, strains USC SE30.1 and NCIMB 2158 displayed a slightly different peak shape for RpmJ-M-H1 and RpmE-M-H2, respectively, with a larger area under the curve and a flatter bump. This resulted from the presence of two close peaks with the same intensity. However, these peaks did not disrupt the signal and the peaks for RpmJ-M-H1 and RpmE-M-H2 could both be accurately detected. The 18 selected peaks corresponded to 9 ribosomal proteins with varying degrees of ionization. They could serve as internal calibration references using the *alignSpectra* function for accurate peak detection. In addition, these monomorphic biomarkers were of utmost interest for species identification and were included in the quality control process of spectra (Additional file 8).

### Selection of 9 polymorphic biomarkers for strain typing and MALDI-Type attribution

**Figure 2:**
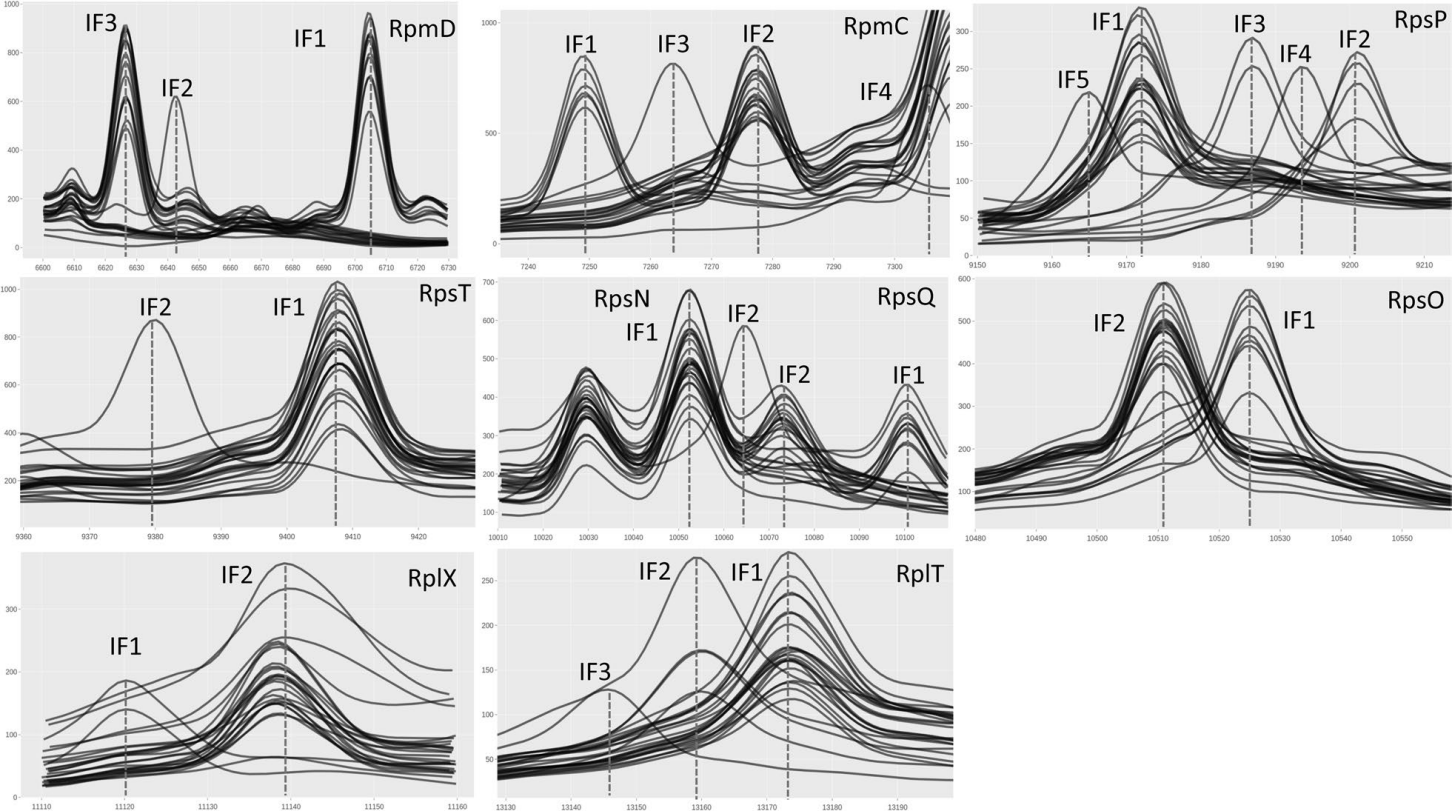
**Characteristic peak shifts used for strain characterization and MALDI-Types definition.** The 24 average spectra are plotted, corresponding to all except one sequenced isolates. Intensity varies from 200 to 1000. The RpmC IF4 (strain NAC SLCC MFF) is present but partially masked by a stronger peak shared by the other 23 isolates. The central figure displays 3 different biomarkers that correspond to 5 different peaks. The first at 10 300 m/z is shared by all isolates in our collection and corresponds to RpsS (species biomarker). The two peaks in the middle correspond to RpsN and the last two peaks correspond to RpsO, isoforms 1 and 2 respectively.

Using our polymorphic biomarker candidates (Additional file 6), we performed visual inspection of the above-mentioned spectra to identify the corresponding peak shifts. We selected 8 polymorphic biomarkers corresponding to ribosomal proteins (i.e., RpmD, RpmC, RpsP, RpsN, RpsO, RpsQ, RplX and RplT) with a molecular weight ranging from 6600 Da to 13 200 Da. An additional polymorphic biomarker, RpsT, was included after screening our collection of 131 isolates (see next paragraph). Indeed, strain Aq8-57 displayed an unexpected peak shift (not correlated to any amino-acid polymorphism observed in the 25 genomes data set). Sanger sequencing of strain Aq8-57 revealed that this peak shift corresponded to a 122A > G mutation in the sequence of the *rpsT* gene leading to a R41K change in the amino-acid sequence. In addition, strain USC RPM 539.1 also displayed a peak shift not previously observed that corresponded to a 35G > A mutation in the sequence of the *rplT* gene leading to a R12K change in the amino-acid sequence. These mutations gave rise to a −27 Da shift observed in the spectra compared to the type strain used as a reference. Therefore, the 9 retained biomarkers displayed varying degrees of polymorphism (Additional file 9) ranging from two to up to five isoforms (IF) (Table 2, Figure 2). An arbitrary number was given to each IF for subsequent analysis. By convention, an IF1 numbering was given for each biomarker of the type strain NCIMB 2154^T^.

**Table 2:** **The retained polymorphic biomarkers.**

Biomarkers	First methionine cleavage	H+	Predicted mass	Observed mass	Delta ppm	Isoform
RpmD	S(2) 91%	1	6700.56	6703.90	498	IF1
			6638.48	6641.21	411	IF2
			6622.48	6625.88	513	IF3
RpmC	M(1) 99%	1	7245.24	7248.41	437	IF1
			7273.26	7275.95	273	IF2
			7259.31	7262.33	416	IF3
			7301.31	7303.94	360	IF4
RpsP	P(2) 98%	2	9168.45	9169.730	139	IF1
			9197.49	9199.03	167	IF2
			9183.47	9184.97	163	IF3
			9190.48	9191.34	93	IF4
			9161.44	9163.107	181	IF5
RpsT	A(2) 97%	1	9404.57	9406.69	225	IF1
			9376	9379.19	225	IF2
RpsN	A(2) 97%	1	10049.41	10051.07	165	IF1
			10061.46	10064.14	266	IF2
RpsQ	M(1) 99%	1	10097.52	10099.99	244	IF1
			10070.50	10071.79	68	IF2
RpsO	M(1) 99%	1	10521.89	10524.30	229	IF1
			10507.82	10510.00	207	IF2
RplX	M(1) 99%	1	11117.46	11119.12	146	IF1
			11135.48	11136.94	131	IF2
RplT	P(2) 88%	1	13171.38	13172.27	67	IF1
			13157.34	13157.79	34	IF2
			13144	13145.75	57	IF3

### Validation of the typing scheme using field isolates

**Figure 3:**
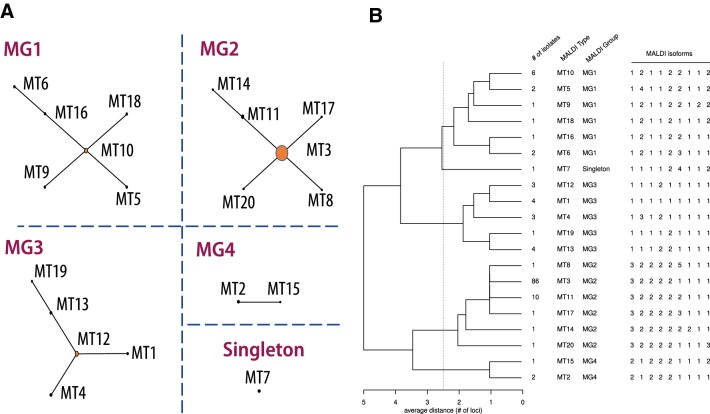
**eBurst network, visual representation of the links between the MALDI-Types and the hierarchical tree based on MALDI-Types isomorphic profile. A** eBurst defines clusters when isolates share 8 out of the 9 polymorphic biomarkers. These clusters may be considered as MALDI-Groups. Each of them contains a central MT, which represents the “founder” MT of the corresponding group. **B** The classification tree, based on average-link hierarchical clustering (average-link method), offers another view of the links existing between MALDI-Types and MALDI-Groups. The distance used for this tree corresponds to the number of differences between each MALDI profile.

To validate the strain typing approach, and in addition to the 24 above-mentioned spectra, we analyzed by MALDI-TOF MS 111 field isolates from worldwide origins and retrieved from a variety of fish species (Additional file 1). Among the field isolates, 56 had previously been typed using MLST [15]. Our strategy based on automated peak detection and assignation to the corresponding IF proved to be very efficient with a success rate of 97%. Only four isolates (P1-40, Aq12-62, Aq12-64 and Aq6-9) out of 111 were not fully typed corresponding to 5 (out of 1176) peak losses (0.42%). We chose an MLST-like strategy by combining IF numbering to produce the corresponding MALDI profile as proposed by Zautner et al. [42], and here referred to as MALDI-Type (MT). Using this scheme, we identified 20 MTs (Additional file 1). Using genome sequence data, one could predict the MT of a strain; for instance, strain TM-KORJJ was predicted to belong to MT3. This approach allowed unambiguous assignment for each isolate as well as the use of visualization and analysis tools developed for MLST such as the eBurst [43] and SplitsTree decompositions [44]. Indeed, using eBurst on our dataset, the 20 MTs could be grouped in 4 clusters based on connection by single-locus-variants (Figure 3A) that can be designated as MALDI-Groups (MGs). Of note, the criterion for the determination of MGs is analogous to the criterion for the determination of clonal complexes (CC) reported in the MLST strategy [45] but does not correspond to the same level of divergence between isolates. Figure 3B is complementary to the graph drawn using eBurst. It depicts a hierarchical clustering (average-link) built from MALDI-Types isomorphic profiles with the corresponding number of isolates.

The MTs and MGs are globally congruent with the core genome-based phylogeny (Figure 1). Indeed, strains USC SP9.1 and USC SE30.1 both belonged to MT6 and were encompassed in clade C. Strains NAC SLCC MFF, FS08(1) and P2-48 were encompassed in clade A and belonged to MTs 5, 9 and 10, respectively. These 3 latter MTs were linked by single locus variants. All of the above-mentioned strains were encompassed in MG1. Strain DPIF 89/0239-1 belonged to MT7, which was the only singleton among all MGs. In the core genome-based phylogeny, strain DPIF 89/0239-1 also appeared distantly related to the other strains encompassed in clade B. Strain P2-27 belonged to MT19 whereas the type strain NCIMB 2154^T^ belonged to MT1 and strain NBRC 15946 belonged to MT4. These three strains were encompassed in MG3. Strain UCD SB2 belonged to MT8 (MG2) and also appeared more distantly related to the other strains encompassed in clade B in the core genome-based phylogeny. Finally, all the other strains belonged to MT3 (MG2) with only two exceptions, strains FC and DPIF 89/023-9 that were the only strains displaying incongruence between MT and core genome-based phylogeny.

Although the 131 strains studied were from worldwide origin, our dataset was not suitable to highlight sound association between the isolation sources and the MT. We tried several statistical analyses (AMOVA and Fisher exact test, data not shown), but each tested variable (i.e., country and year of isolation, host fish species) was drastically correlated with each other indicating that these variables are not truly independent. For instance, a high correlation between fish hosts and countries was obvious. However, and in accordance with our previous MLST study [15], we observed trends between the geographical origin of the strains and the MT. For instance, the 4 isolates belonging to MT1 and the 3 isolates belonging to MT4 all originated from Japan. In addition, these two MTs belong to the same MG3 and are linked by Double Locus Variants (DLV). The two isolates belonging to MT2 originated from California. The two isolates belonging to MT5 originated from Italy and Malta, two neighboring countries. The two isolates belonging to MT6 originated from Spain. The three isolates belonging to MT12 originated from Tasmania. In addition, 45 out of the 50 isolates from France belonged to MT3. On the other hand, strains from the same geographical origin could belong to different MTs (e.g., the 10 Tasmanian isolates belonged to 4 different MTs whereas the 5 Italian strains each belonged to a different MT). Of importance, strains retrieved from the same host fish species could belong to different, unrelated MTs (e.g., the 74 strains retrieved from *Dicentrarchus labrax* belonged to 5 different MTs).

### Evaluation of reproducibility, repeatability and alternative sample preparations for MALDI-TOF MS

In order to evaluate reproducibility and repeatability, the *T. maritimum* type strain, the 22 genome-sequenced *T. maritimum* strains and the type strains of the 23 other *Tenacibaculum* species included in this study were subjected to multiple (*n* ≥ 3), independent, MALDI-TOF MS data acquisitions (one acquisition corresponding to an average spectrum of four technical replicates) using the ethanol/formic acid extraction procedure from fresh bacterial culture. Using this set of reference strains, the accuracy was 100%. In addition, laboratories may use different procedures [46] for MALDI-TOF MS analysis (from sample preparation to data processing and interpretation). To address these issues, we tested several sample preparation protocols. The quickest and easiest way to acquire MALDI-TOF MS data from a bacterium is direct transfer method. We evaluated this protocol with 104 bacterial isolates arbitrarily sampled among the 135 above-mentioned isolates. Among these, only 9 strains were not fully typed (8.65%) because of about 1% peak loss. We also assessed our method with ethanol-fixed bacteria, a strategy that could facilitate the MALDI-TOF MS typing of isolates from distant locations. We evaluated the effects of long term (up to 1 month) ethanol conservation followed by the regular protein-extraction protocol. Fifty-one out of 62 isolates (82%) were fully typed after 7 to 15 days ethanol storage. However, only 15 out of 25 isolates (60%) were fully typed after 16 to 26 days ethanol storage. Hence, the conservation time significantly affected typing reliability. Of importance, IF assignments were always congruent for the same sample whatever the preparation method used.

### A web-based tool for MALDI-Type assignment

We felt that a web-based tool for MT assignment of *T. maritimum* strains would facilitate data interpretation and help comparisons at a global scale in the same way as previously proposed for MLST schemes [47]. Hence, we developed a friendly application named MALDIquantTypeR for *Tenacibaculum* MALDI-TOF data analysis that contains 3 tools. Firstly, in order to avoid misinterpretation with spectra from bacteria that do not belong to the species *T. maritimum*, we added reference spectra from 24 out of the 29 described *Tenacibaculum* species, including all the fish-pathogenic or fish-associated species (i.e., *T. dicentrarchi*, *T. discolor*, “*T. finnmarkense*”, *T. gallaicum*, *T. ovolyticum* and *T. soleae*). Each reference spectrum is composed of a peak list obtained with *Tenacibaculum* type strains following Bruker’s recommendations (> 20 independent acquisitions). We used stringent parameters and kept peaks with a signal/noise ratio > 3. Thus, each *Tenacibaculum* species is defined by 20 to 60 peaks. When raw spectra from an unidentified sample are uploaded in the application, a peak list is produced using the same stringent parameters and each retained peak is compared to the reference peak file. A peak is considered matching the reference if the difference between two values is less than 700 ppm. Then, the number of matching peaks between the sample and each type strain is computed. The output is a bar-plot providing the percentage of matching peaks with the references corresponding to the 24 *Tenacibaculum* species included so far. For instance, all *T. maritimum* isolates used in this study display at least 50% of common peaks with the *T. maritimum* type strain (vs < 20% with the type strains of other *Tenacibaculum* species). This first tool suggests a taxonomic affiliation and only selects the spectra likely belonging to the species *T. maritimum.* The second tool uses the 18 *T. maritimum* specific monomorphic biomarkers previously defined. It is based on the same peak matching strategy to accurately identify a spectrum as belonging to the species *T. maritimum.* In addition, it also provides a valuable measure of spectra data quality (Additional file 8). The third tool is the typing method itself that identifies the IF of the 9 polymorphic biomarkers and provides the isomorphic profile and the resulting combination corresponding to the MT. This profile can further be processed in the same way as an MLST profile. The MALDIquantTypeR application is available online [23] hosted by the Migale platform at INRAE Jouy-en-Josas.

## Discussion

The fast development of aquaculture faces an array of sanitary issues, causing important economic losses and impacting the environment and animal welfare [1, 2]. The rapid detection of pathogens and the continuous monitoring of circulating bacterial genotypes in space and time is a prerequisite for the implementation of rational control measures [48]. International and cross-sector surveillance of pathogens requires strain typing methods that must be rapid, accurate, resolutive, reproducible and affordable, and that must enable the exchange of molecular typing data via the Internet [1].

Tenacibaculosis is an ulcerative disease affecting many marine fish species of commercial interest worldwide and *T. maritimum* is considered the main causative agent of tenacibaculosis in wild and cultured fish [5]. Different typing methods for epidemiological investigations of *T. maritimum* have been proposed [9]. However, they do not fit all the above-mentioned recommendations. For instance, the different serotyping schemes that have been proposed [10, 11, 49] appear to be unrelated and poorly discriminatory (only three serotypes have been reported to date and some strains display cross-reactivity). Moreover, conventional serology is costly, labor-intensive and requires significant technical expertise and the use of animals to raise anti-sera. On the other hand, an MLST scheme has been proposed [15] and proved to be a powerful discriminating tool for isolate identification and strain typing. However, classical MLST is also costly, labor-intensive and time consuming. Whole genome sequencing represents the “ultimate” typing methodology in terms of discriminatory power. However, it is not yet suitable for real time monitoring of large collections of bacterial isolates and is mostly used for retrospective analysis.

In this study, we first used genomic comparisons of 25 strains including 22 newly draft-sequenced genomes to draw a global picture of the genomic diversity of the species. *Tenacibaculum maritimum* appears as a cohesive bacterial species which strains are characterized by: (i) a high genomic identity (ANI > 98%); (ii) a similar genome size (~3,4 Mb); and (iii) a moderate level of nucleotide divergence (maximum 1.52% in pairwise core-genome sequence comparisons) while typical bacterial species can exhibit up to ~5% nucleotide divergence [50]. In addition, a major contribution of recombination (r/m ≥ 7) in the evolutionary process of the species was observed, reminiscent of the situation observed in another fish-pathogenic species of the family *Flavobacteriaceae*, *Flavobacterium. psychrophilum* [35]. Our data set encompasses two fully PacBio-assembled genomes (i.e., NCIMB 2154^T^ and TM-KORJJ) that are perfectly collinear, pointing to a similar chromosomal organization without major genomic rearrangements.

Overall, our results based on 25 genomes are not only in good accordance with the conclusions drawn from our previous analysis using a 11 loci-based MLST data set [15], but they provide unprecedented details on genome content and organization. Tentative phylogenomic tree reconstructions using different methods are congruent and support the division in three main clades (A, B and C). In addition to core-genome genes similarities, clades A and C share some common genomic features. For example, the RplU encoding gene version is obviously different between clades A/C and clade B strains (209 amino-acid long in clade A and C strains vs a 161 amino-acid long in clade B strains). The *metE* gene, encoding methionine synthase, is full length in clade B strains but pseudogenized in clades A/C strains suggesting a non-functional methionine biosynthesis pathway in the latter. Additional clade-specific features were identified. For example, the two clade C strains display non-functional, frame shifted versions of genes of which full-length versions are present in all the other genomes examined (e.g., strain NCIMB 2154^T^ locus tags: *MARIT_0127*, *MARIT_0229*, *MARIT_0258*, *MARIT _0523*, *MARIT _0991*, *MARIT_1553*, *MARIT_1615*, *MARIT_1833*, *MARIT_1972*, *MARIT_2492*, *MARIT_2635* and *MARIT_2635*). These observations performed on a limited number of genomes may face some exceptions (e.g., due to gene shuffling by homologous recombination). However, the presence of such gene remnants in the genome of some strains argues for genome reduction trends as frequently observed in bacterial pathogens [51]. Strikingly, most of the variable-genome encoding genes are located in genomic islands, some restricted to a single strain while others are shared between several strains. It is therefore tempting to speculate that some islands provide a fitness advantage such as the one containing heavy metal resistance genes identified in strain FC to face an environment polluted by heavy metals. Indeed, important copper concentrations in sediments because of disposal of copper mine tailings has been documented for bays of northern Chile, the region where strain FC was isolated from. A copper-resistant *Vibrio* sp. strain was retrieved from cultured scallop [52] from the same geographical area suggesting convergent evolutionary mechanisms for heavy metal resistance in these phylogenetically unrelated marine bacteria.

In addition to providing a global picture of the genomic diversity, the use of genomic data has been a key step in the MALDI-TOF MS scheme proposed in this study by allowing a better understanding of the spectral composition. The scheme follows Sauget et al. [46] MALDI-TOF MS recommendations by using the genomic information for selecting relevant biomarkers, by choosing ribosomal proteins that should be unambiguously identified within the spectra and by focusing exclusively on pic shifts for the typing purpose. Indeed, we were able to retrieve from the spectra 18 out of 54 in silico-identified ribosomal proteins. The total number of biomarkers (18 monomorphic and 9 polymorphic) corresponds to about one-third of the detected peaks. In addition, most of these peaks display high intensity, even at both ends of the spectra. Combining genome mining and visual exploration of the spectra, we linked each noteworthy peak shift with the corresponding polymorphism in genomic sequences (Additional file 9). Strikingly, the 9 polymorphic biomarkers and their corresponding isoforms (25 in total) were defined from only 10 strains (out of 25 sequenced isolates), reflecting the cohesive nature of the *T. maritimum* species. The strategy based on highly relevant biomarkers and their selection based on stringent criteria proved to be particularly effective. Using 111 field isolates and the ethanol/formic acid extraction procedure, automated peak detection displayed a very high level of success rate (> 99.5% of the polymorphic biomarkers identified). Because fluctuations in MALDI-TOF MS results might be caused by the use of different bacterial growth conditions, sample preparation procedures or matrix used [46], we evaluated the robustness and reliability of the proposed typing scheme by using biological and technical replicates as well as alternative sample preparations (i.e., direct transfer method and ethanol/formic extraction after ethanol conservation). As detailed above, the results are still very good when alternative sample preparation procedures are used, though the success rate may be lower, in particular using long-term ethanol conservation. The observed peak detection failures may be attributed to random ion suppression intrinsically linked to the analogical nature of the spectra [46]. This concern may, however, be simply addressed by data re-acquisition (Additional file 8).

Because peak shifts are the direct consequence of a non-synonymous mutation in a ribosomal protein, a peak shift may be considered as an allelic change as proposed by Zautner et al. [42]. We therefore treated peaks shifts as a combination of alleles. A single strain would thus display a unique combination that could be considered as a MALDI-type (MT). These MTs are therefore the equivalent of the sequence types (STs) or electrophoretic types (ETs) resulting from the MLST or MLEE schemes, respectively. In the same way as the latter two schemes, the proposed Multi Peak Shift Typing (MPST) scheme for *T. maritimum* could be enriched in the future by considering additional biomarkers and/or by the identification of additional IF in the retained biomarkers. New, yet unobserved, combinations of isoforms may also be identified in the future. The definition of new MTs will follow the same incremental process as previously proposed for MLST schemes.

Applied to our collection of *T. maritimum* isolates, this method identified 20 MTs grouped in 4 MGs. Because our goal was to propose and to evaluate a MPST scheme dedicated to the typing of *T. maritimum* strains, we used isolates from broad geographical, temporal and host-fish species origins to cover as much of the species diversity as possible. We are aware of the inherent bias of such a sampling choice. Indeed, the variables (i.e. country, year of isolation, host fish species) were highly correlated with each others; therefore, this dataset is not suitable for highlighting sound associations between isolation sources and MTs. However, we observed trends between the geographical origin of the strains and the MT as previously reported using MLST data [15]. In addition, and in line with previous MLST-based conclusions, our analysis revealed no trace of long-distance dissemination of *T. maritimum* that could be linked to the international trade of fish or eggs.

MPST schemes based on MALDI-TOF MS data may prove suitable for large-scale epidemiological studies for a number of reasons: (i) the approach is similar to MLST, and the tools used to analyze MLST data can also be used on MPST data; (ii) MALDI-TOF acquisitions are much cheaper and faster (about 20 minutes for a whole MALDI-TOF plate acquisition) than sequencing- or PCR-based MLST; (iii) 96 samples can be deposited on the same MALDI-TOF plate; and (iv) the scheme can be transposed to any bacterial species that contains polymorphic ribosomal proteins or any noteworthy polymorphic biomarker. Indeed, the peak shifts caught by MALDI-TOF MS are highly correlated to the genotype as defined in any MLST analysis. MALDI-TOF spectra databases and processing tools can be easily exported on the Internet as a webtool application in the same way as MLST web-based tools (e.g., PubMLST.org). MALDIquantTypeR is one example of such application available online [23] as well as other dedicated websites such as the Mass Spectrometry Identification platform [53]. Large-scale studies could easily benefit from worldwide data collection. We have shown that ethanol (at least short-term) conservation can be used for MALDI-TOF identification and typing. This method also suppresses biological hazard and should facilitate international transportation of bacterial samples. Other solutions may be designed for international collaboration, such as direct shipment of MALDI-TOF plates with fixed biological material, ready for acquisition. MPST analysis could also be interesting in local surveys monitoring bacterial populations in particularly sensitive geographical areas.

## Supplementary information

**Additional file 1.** ***T. maritimum*** **isolates used in this study and their corresponding MALDI-Types.**

**Additional file 2. Average Nucleotide Identity (ANI) of pairwise comparison of the** ***T. maritimum*** **isolates.**

**Additional file 3. Parsimony based phylogenetic tree.** The tree is based on the alignment made by Snippy. It is reconstructed using the parsimony method as implemented in *dnapars* (Phylip package v3.6). The bootstrap support of each branch is computed from 100 bootstrap replicates. The three clades designated A, B, and C are labeled and delineated by vertical bars.

**Additional file 4. Maximum likelihood, Gubbins-based phylogenetic tree.** The tree was obtained from the whole genome alignment of 25 *T. maritimum* strains at the fifth and final iteration of Gubbins. Statistical support of nodes is indicated. The three clades designated A, B, and C are labeled and delineated by vertical bars.

**Additional file 5. Overview of recombination tracts between two pairs of*T. maritimum*isolates. SNPs and recombination tracts between two closely related isolates.** In 5A the strains compared are USC SP9.1 and USC SE30.1. In 5B the strains compared are FS08(1) and P2−48. (Upper) Positions of the SNPs along the genomes. SNP index is reset every 100 SNPs for this representation. Each dot corresponds to one SNP in the comparison between the two considered isolates. Colors distinguish two types of polymorphism: in blue, polymorphism observed only between the two considered genomes; in red, polymorphism also observed among the other sequenced genomes. Areas in gray correspond to regions not covered by our alignments. SNPs in regions where probability is < 0.5 (i.e. outside predicted recombination tracts) are represented by open symbols (blue circles). (Lower) Probability of recombination tract as computed with the HMM. Estimation of the  % of genome in recombination tracts is 15.8 and 19.1 for (A) and (B), respectively. Estimation of the average length of recombination tracts is 885 bp and 328 bp. for (A) and (B), respectively. Estimation of the average nucleotide diversity inside recombination tracts is 0.013/bp and 0.014/bp for (A) and (B), respectively. Estimation of the average nucleotide diversity outside recombination tracts is 9.9e-5/bp and 3.5e-4/bp for (A) and (B), respectively. Estimation of the number of SNPs inside recombination tracts is 5266 and 7013 for (A) and (B), respectively. Estimation of the number of SNPs outside recombination tracts is 216 and 737 for (A) and (B), respectively. Estimation of the number of SNPs due to mutations (extrapolated from non − recombined regions) is 256 and 910 for (A) and (B), respectively. Estimation of the ratio r/m is 20.6 and 7.7 for (A) and (B), respectively.

**Additional file 6. Heatmap displaying the diversity of ribosomal protein weights.** Lines correspond to strains and columns correspond to ribosomal proteins in ascending order by weight (from left to right). White lines indicate no variation of the weight of the corresponding proteins (i.e., monomorphic proteins). In purple, proteins with weight higher than the mean and in orange proteins with weight lower than the mean (i.e., polymorphic proteins).

**Additional file 7. Monomorphic biomarker peaks. Screenshots of the 18 conserved peaks produced by 9 ribosomal monomorphic proteins with several degrees of ionization**. The m/z values are highlighted by dotted lines and cover the entire spectrum. The red curve corresponds to the *T. maritimum* type strain average spectra. For each peak, the corresponding ribosomal protein is indicated with the degree of ionization (H1, H2 and H3 corresponding to 1, 2 and 3 H^+^) and the presence (M) or absence (m) of the first methionine. Color code: red line for the *T. maritimum* type strain NCIMB 2154^T^ and black for the sequenced *T. maritimum* isolates.

**Additional file 8. Quality control and*T. maritimum*species identification.** The full dataset is composed of representatives of 24 *Tenacibaculum* species including 135 isolates belonging to the species *T. maritimum.* It encompasses 476 independent acquisitions (one acquisition corresponds to an average spectrum of several technical replicates) corresponding to 5102 spectra including technical and biological replicates. This dataset was divided into two groups: the positive control group (*T. maritimum* isolates) and the negative control group (the type strains of 23 other *Tenacibaculum* species). In order to confirm that an isolate belongs to the species *T. maritimum*, the spectra were scanned to identify the 18 *T. maritimum* monomorphic biomarkers. However, some of these biomarkers could be absent from a number of *T. maritimum* strains. Reciprocally, strains that do not belong to the *T. maritimum* species may possess some *T. maritimum* monomorphic biomarkers. In order to set up a *T. maritimum* species identity threshold, a value corresponding to the number of monomorphic biomarkers identified in a single sample was computed. The monomorphic biomarkers frequency plot obtained shows a bimodal distribution (Figure A). All samples with a score above 60% correspond to *bona fide T. maritimum* isolates while all samples with a score below 25% belong to other *Tenacibaculum* species. True and false positives correspond to isolates correctly and incorrectly identified as *T. maritimum* (TP and FP), respectively. On the other hand, true and false negatives correspond to correctly and incorrectly rejected isolates (TN and FN, respectively). Positive isolates correspond to those having an identity value above a defined threshold. Using the full dataset, the number of TP and FP and the number of TN and FN were counted. The accuracy of the tool [i.e., (TP + TN)/(TP + TN + FP + FN)] was then computed by increasing the threshold value from 0% to 100% by a 0.1% step. One could observe than the accuracy varies from 0% to 100% and is maximal (i.e., above 97%) between 25% and 60% of threshold value (Figure B). It is therefore proposed that a 60% threshold value safely identifies isolates as belonging to the *T. maritimum* species. Using this 60% threshold value, only 10 false negatives (i.e., *bona fide T. maritimum* isolates discarded) and 0 false positive (i.e., not *T. maritimum* isolates) were found out of 476 acquisitions. Among the 10 false negatives, 3 resulted from the extraction protocol, 3 from the Direct Deposit with Formic Acid (DDFA) protocol and 4 from the ethanol conservation protocol (see section “Testing alternative sample preparation for MALDI-TOF MS”). One can hypothesize that these rare false negatives correspond to technical problems. Indeed, by performing new spectra acquisitions on the 3 isolates previously analyzed by the extraction protocol, the identification score reached at least 88%, far above the safe threshold value of 60%, demonstrating that the original spectra were likely faulty. Finally, spectra from other *Tenacibaculum* species all had a score below 23%, far below the confidence threshold.

**Additional file 9. Amino-acid polymorphism of the 9 retained biomarkers.** Protein sequence alignments of the 9 ribosomal proteins selected as polymorphic biomarkers and their corresponding isoform (IF).

## Data Availability

All genome sequencing data have been deposited in the European Nucleotide Archive (Accession numbers: GCA_902705265, GCA_902705275, GCA_902705285, GCA_902705305, GCA_902705315, GCA_902705345, GCA_902705355, GCA_902705365, GCA_902705375, GCA_902705385, GCA_902705395, GCA_902705415, GCA_902705425, GCA_902705435, GCA_902705445, GCA_902705465, GCA_902705495, GCA_902705515, GCA_902705525, GCA_902705535, GCA_902705555 and GCA_902705565).

## References

[1] Bayliss SC, Verner-Jeffreys DW, Bartie KL, Aanensen DM, Sheppard SK, Adams A, Feil EJ (2017). The promise of whole genome pathogen sequencing for the molecular epidemiology of emerging aquaculture pathogens. Front Microbiol.

[2] FAO (2016) The State of World Fisheries and Aquaculture. FAO, Rome. http://www.fao.org/3/a-i5555e.pdf

[3] Adam KE, Gunn GJ (2017). Social and economic aspects of aquatic animal health. Rev Sci Tech.

[4] Rodgers CJ, Mohan CV, Peeler EJ (2011). The spread of pathogens through trade in aquatic animals and their products. Rev Sci Tech.

[5] Avendaño-Herrera R, Toranzo AE, Magariños B (2006). Tenacibaculosis infection in marine fish caused by *Tenacibaculum maritimum*: a review. Dis Aquat Organ.

[6] Gourzioti E, Kolygas M, Athanassopoulou F, Babili V (2018). Tenacibaculosis in aquaculture farmed marine fish. J Hell Vet Med Soc.

[7] Rahman T, Suga K, Kanai K, Sugihara Y (2014). Biological and serological characterization of a non-gliding strain of *Tenacibaculum maritimum* isolated from a diseased puffer fish *Takifugu rubripes*. Fish Pathol.

[8] Avendaño-Herrera R, Núñez S, Barja JL, Toranzo AE (2008). Evolution of drug resistance and minimum inhibitory concentration to enrofloxacin in *Tenacibaculum maritimum* strains isolated in fish farms. Aquac Int.

[9] Fernández-Álvarez C, Santos Y (2018). Identification and typing of fish pathogenic species of the genus *Tenacibaculum*. Appl Microbiol Biotechnol.

[10] Avendaño-Herrera R, Magariños B, López-Romalde S, Romalde JL, Toranzo AE (2004). Phenotypic characterization and description of two major O-serotypes in *Tenacibaculum maritimum* strains from marine fishes. Dis Aquat Organ.

[11] Avendaño-Herrera R, Magarinos B, Morinigo M, Romalde J, Toranzo A (2005). A novel O-serotype in *Tenacibaculum maritimum* strains isolated from cultured sole (*Solea senegalensis*). Bull Eur Assoc Fish Pathol.

[12] Avendaño-Herrera R, Rodríguez J, Magariños B, Romalde JL, Toranzo AE (2004). Intraspecific diversity of the marine fish pathogen *Tenacibaculum maritimum* as determined by randomly amplified polymorphic DNA-PCR. J Appl Microbiol.

[13] Piñeiro-Vidal M, Pazos F, Santos Y (2008). Fatty acid analysis as a chemotaxonomic tool for taxonomic and epidemiological characterization of four fish pathogenic *Tenacibaculum* species. Lett Appl Microbiol.

[14] Fernández-Álvarez C, Torres-Corral Y, Santos Y (2018). Comparison of serological and molecular typing methods for epidemiological investigation of *Tenacibaculum* species pathogenic for fish. Appl Microbiol Biotechnol.

[15] Habib C, Houel A, Lunazzi A, Bernardet J-F, Olsen AB, Nilsen H, Toranzo AE, Castro N, Nicolas P, Duchaud E (2014). Multilocus sequence analysis of the marine bacterial genus *Tenacibaculum* suggests parallel evolution of fish pathogenicity and endemic colonization of aquaculture systems. Appl Environ Microbiol.

[16] Avendaño-Herrera R, Irgang R, Sandoval C, Moreno-Lira P, Houel A, Duchaud E, Poblete-Morales M, Nicolas P, Ilardi P (2016). Isolation, characterization and virulence potential of *Tenacibaculum dicentrarchi* in salmonid cultures in Chile. Transbound Emerg Dis.

[17] Klakegg Ø, Abayneh T, Fauske AK, Fülberth M, Sørum H (2019). An outbreak of acute disease and mortality in Atlantic salmon (*Salmo salar*) post-smolts in Norway caused by *Tenacibaculum dicentrarchi*. J Fish Dis.

[18] Olsen AB, Gulla S, Steinum T, Colquhoun DJ, Nilsen HK, Duchaud E (2017). Multilocus sequence analysis reveals extensive genetic variety within *Tenacibaculum* spp. associated with ulcers in sea-farmed fish in Norway. Vet Microbiol.

[19] Fernández-Álvarez C, Torres-Corral Y, Saltos-Rosero N, Santos Y (2017). MALDI-TOF mass spectrometry for rapid differentiation of *Tenacibaculum* species pathogenic for fish. Appl Microbiol Biotechnol.

[20] Pérez-Pascual D, Lunazzi A, Magdelenat G, Rouy Z, Roulet A, Lopez-Roques C, Larocque R, Barbeyron T, Gobet A, Michel G, Bernardet J-F, Duchaud E (2017). The complete genome sequence of the fish pathogen *Tenacibaculum maritimum* provides insights into virulence mechanisms. Front Microbiol.

[21] Bridel S, Olsen A-B, Nilsen H, Bernardet J-F, Achaz G, Avendaño-Herrera R, Duchaud E (2018). Comparative genomics of *Tenacibaculum dicentrarchi* and “*Tenacibaculum finnmarkense*” highlights intricate evolution of fish-pathogenic species. Genome Biol Evol.

[22] Hou TY, Chiang-Ni C, Teng SH (2019). Current status of MALDI-TOF mass spectrometry in clinical microbiology. J Food Drug Anal.

[23] MALDIquantTypeR by S. Bridel. http://genome.jouy.inra.fr/shiny/maldiquanttyper/. Accessed 10 Apr 2020

[24] Wattam AR, Abraham D, Dalay O, Disz TL, Driscoll T, Gabbard JL, Gillespie JJ, Gough R, Hix D, Kenyon R, Machi D, Mao C, Nordberg EK, Olson R, Overbeek R, Pusch GD, Shukla M, Schulman J, Stevens RL, Sullivan DE, Vonstein V, Warren A, Will R, Wilson MJC, Yoo HS, Zhang C, Zhang Y, Sobral BW (2014). PATRIC, the bacterial bioinformatics database and analysis resource. Nucleic Acids Res.

[25] Médigue C, Calteau A, Cruveiller S, Gachet M, Gautreau G, Josso A, Lajus A, Langlois J, Pereira H, Planel R, Roche D, Rollin J, Rouy Z, Vallenet D (2017). MicroScope-an integrated resource for community expertise of gene functions and comparative analysis of microbial genomic and metabolic data. Brief Bioinform.

[26] Richter M, Rosselló-Móra R (2009). Shifting the genomic gold standard for the prokaryotic species definition. Proc Natl Acad Sci U S A.

[27] Pritchard L (2020) https://github.com/widdowquinn/pyani. Accessed 10 Apr 2020

[28] MicroScope home-MaGe: microbial genome annotation & analysis platform-MicroScope-web interface system & specialized databases for (re)annotation and analysis of microbial genomes. https://mage.genoscope.cns.fr/microscope/home/index.php. Accessed 10 Apr 2020

[29] Vallenet D, Calteau A, Dubois M, Amours P, Bazin A, Beuvin M, Burlot L, Bussell X, Fouteau S, Gautreau G, Lajus A, Langlois J, Planel R, Roche D, Rollin J, Rouy Z, Sabatet V, Médigue C (2019). MicroScope: an integrated platform for the annotation and exploration of microbial gene functions through genomic, pangenomic and metabolic comparative analysis. Nucleic Acids Res.

[30] Gautreau G (2020) https://github.com/ggautreau/PPanGGOLiN. Accessed 10 Apr 2020

[31] Seemann T (2020) https://github.com/tseemann/snippy; Accessed 10 Apr 2020

[32] Croucher NJ, Page AJ, Connor TR, Delaney AJ, Keane JA, Bentley SD, Parkhill J, Harris SR (2015). Rapid phylogenetic analysis of large samples of recombinant bacterial whole genome sequences using Gubbins. Nucleic Acids Res.

[33] PHYLIP Home Page. http://evolution.genetics.washington.edu/phylip.html. Accessed 10 Apr 2020

[34] Felsenstein J (1985). Confidence limits on phylogenies: an approach using the bootstrap. Evolution.

[35] Duchaud E, Rochat T, Habib C, Barbier P, Loux V, Guérin C, Dalsgaard I, Madsen L, Nilsen H, Sundell K, Wiklund T, Strepparava N, Wahli T, Caburlotto G, Manfrin A, Wiens GD, Fujiwara-Nagata E, Avendaño-Herrera R, Bernardet J-F, Nicolas P (2018). Genomic diversity and evolution of the fish pathogen *Flavobacterium psychrophilum*. Front Microbiol.

[36] Mellmann A, Cloud J, Maier T, Keckevoet U, Ramminger I, Iwen P, Dunn J, Hall G, Wilson D, Lasala P, Kostrzewa M, Harmsen D (2008). Evaluation of matrix-assisted laser desorption ionization-time-of-flight mass spectrometry in comparison to 16S rRNA gene sequencing for species identification of nonfermenting bacteria. J Clin Microbiol.

[37] TermiNator. https://bioweb.i2bc.paris-saclay.fr/terminator3/. Accessed 10 Apr 2020

[38] Bioconductor-Home. https://bioconductor.org/. Accessed 10 Apr 2020

[39] Du P, Kibbe WA, Lin SM (2006). Improved peak detection in mass spectrum by incorporating continuous wavelet transform-based pattern matching. Bioinformatics.

[40] Ryzhov V, Fenselau C (2001). Characterization of the protein subset desorbed by MALDI from whole bacterial cells. Anal Chem.

[41] Martinez A, Traverso JA, Valot B, Ferro M, Espagne C, Ephritikhine G, Zivy M, Giglione C, Meinnel T (2008). Extent of N-terminal modifications in cytosolic proteins from eukaryotes. Proteomics.

[42] Zautner AE, Masanta WO, Weig M, Groß U, Bader O (2015). Mass spectrometry-based phyloproteomics (MSPP): a novel microbial typing Method. Sci Rep.

[43] Feil EJ, Li BC, Aanensen DM, Hanage WP, Spratt BG (2004). eBURST: inferring patterns of evolutionary descent among clusters of related bacterial genotypes from multilocus sequence typing data. J Bacteriol.

[44] Huson DH, Bryant D (2006). Application of phylogenetic networks in evolutionary studies. Mol Biol Evol.

[45] Urwin R, Maiden MCJ (2003). Multi-locus sequence typing: a tool for global epidemiology. Trends Microbiol.

[46] Sauget M, Valot B, Bertrand X, Hocquet D (2017). Can MALDI-TOF mass spectrometry reasonably type bacteria?. Trends Microbiol.

[47] Jolley KA, Bray JE, Maiden MCJ (2018). Open-access bacterial population genomics: BIGSdb software, the PubMLST.org website and their applications. Welcome Open Res.

[48] van Belkum A, Tassios PT, Dijkshoorn L, Haeggman S, Cookson B, Fry NK, Fussing V, Green J, Feil E, Gerner-Smidt P, Brisse S, Struelens M, European Society of Clinical Microbiology and Infectious Diseases (ESCMID) Study Group on Epidemiological Markers (ESGEM) (2007). Guidelines for the validation and application of typing methods for use in bacterial epidemiology. Clin Microbiol Infect.

[49] Wakabayashi H (1984). *Flexibacter* infection in cultured marine fish in Japan. Helgol Meeresunters.

[50] Kim M, Oh H-S, Park S-C, Chun J (2014). Towards a taxonomic coherence between average nucleotide identity and 16S rRNA gene sequence similarity for species demarcation of prokaryotes. Int J Syst Evol Microbiol.

[51] Weinert LA, Welch JJ (2017). Why might bacterial pathogens have small genomes?. Trends Ecol Evol.

[52] Miranda CD, Rojas R (2006). Copper accumulation by bacteria and transfer to scallop larvae. Mar Pollut Bull.

[53] Normand AC, Becker P, Gabriel F, Cassagne C, Accoceberry I, Gari-Toussaint M, Hasseine L, De Geyter D, Pierard D, Surmont I, Djenad F, Donnadieu JL, Piarroux M, Ranque S, Hendrickx M, Piarroux R (2017). Validation of a new Web application for identification of fungi by use of Matrix-Assisted Laser Desorption Ionization-Time of Flight Mass Spectrometry. J Clin Microbiol.

[54] Institut de Biologie Intégrative de la Cellule https://www.i2bc.paris-saclay.fr/. Accessed 10 Apr 2020

[55] CEA (2018) About Genoscope: CEA François Jacob Inst. Biol. http://www.cea.fr/drf/ifrancoisjacob/english/Pages/Departments/Genoscope/About-Genoscope.aspx. Accessed 10 Apr 2020

[56] Migale platform. https://migale.inra.fr/. Accessed 10 Apr 2020

