# Supplementary material for: Genetic diversity and population structure of Tenacibaculum maritimum, a serious bacterial pathogen of marine fish: from genome comparisons to high throughput MALDI-TOF typing
Source: Vet Res. 2020 May 7;51:60. doi: 10.1186/s13567-020-00782-0 (PMC7204230; doi:10.1186/s13567-020-00782-0)
Supplement: Supplementary file 1 — Additional file 1. T. maritimum isolates used in this study and their corresponding MALDI-Types. [file 13567_2020_782_MOESM1_ESM.docx]

** denotes a sample of uncertain origin*

NT equivalent to non-typable, used for missing biomarkers

List of contributors

List of abbreviations
