# Supplementary figures and images for: Genetic diversity and population structure of Tenacibaculum maritimum, a serious bacterial pathogen of marine fish: from genome comparisons to high throughput MALDI-TOF typing
Source: Vet Res. 2020 May 7;51:60. doi: 10.1186/s13567-020-00782-0 (PMC7204230; doi:10.1186/s13567-020-00782-0)

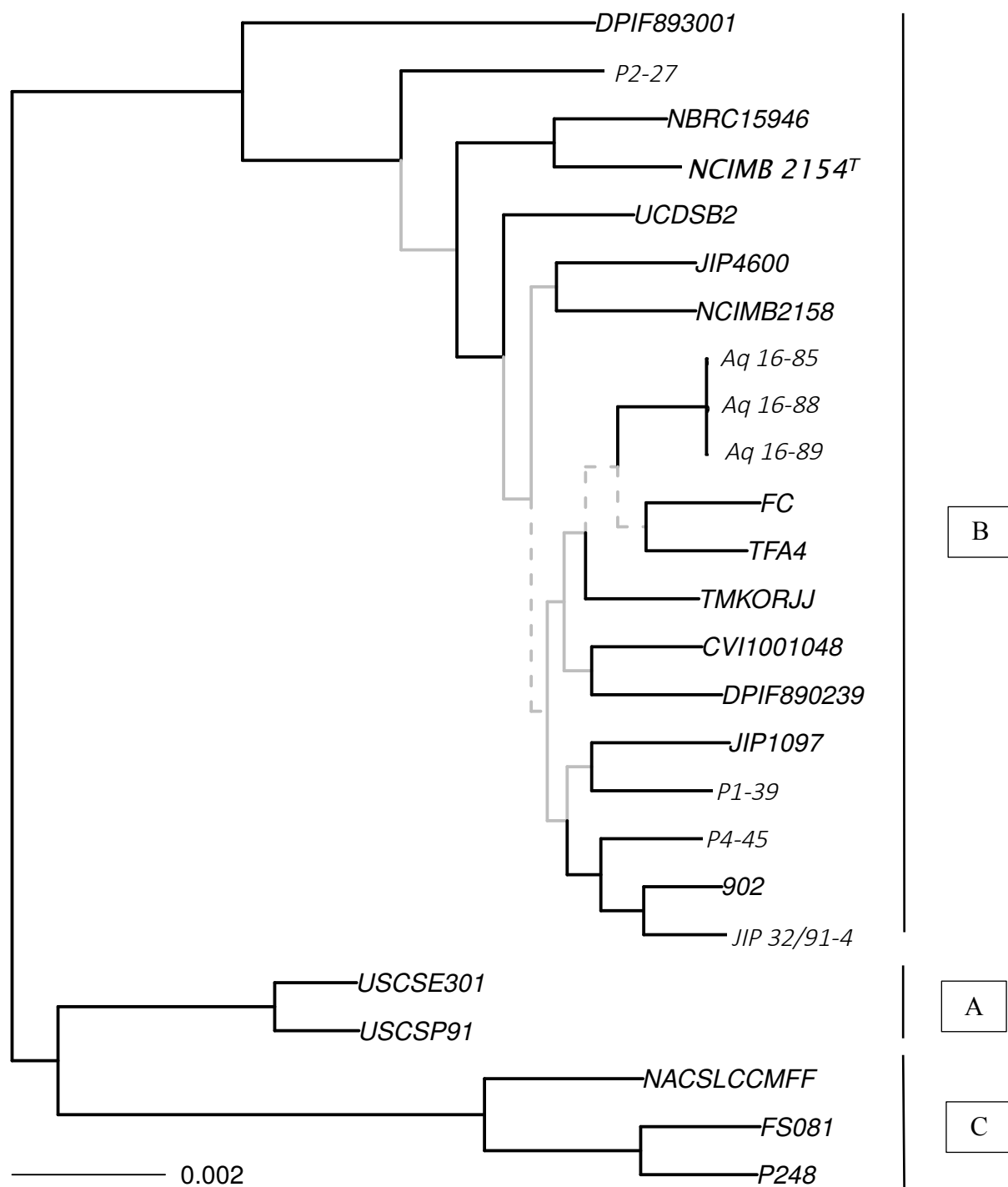

Supplement: Supplementary file 3 — Additional file 3. Parsimony based phylogenetic tree. The tree is based on the alignment made by Snippy. It is reconstructed using the parsimony method as implemented in dnapars (Phylip package v3.6). The bootstrap support of each branch is computed from 100 bootstrap replicates. The three clades designated A, B, and C are labeled and delineated by vertical bars. [file 13567_2020_782_MOESM3_ESM.pdf]

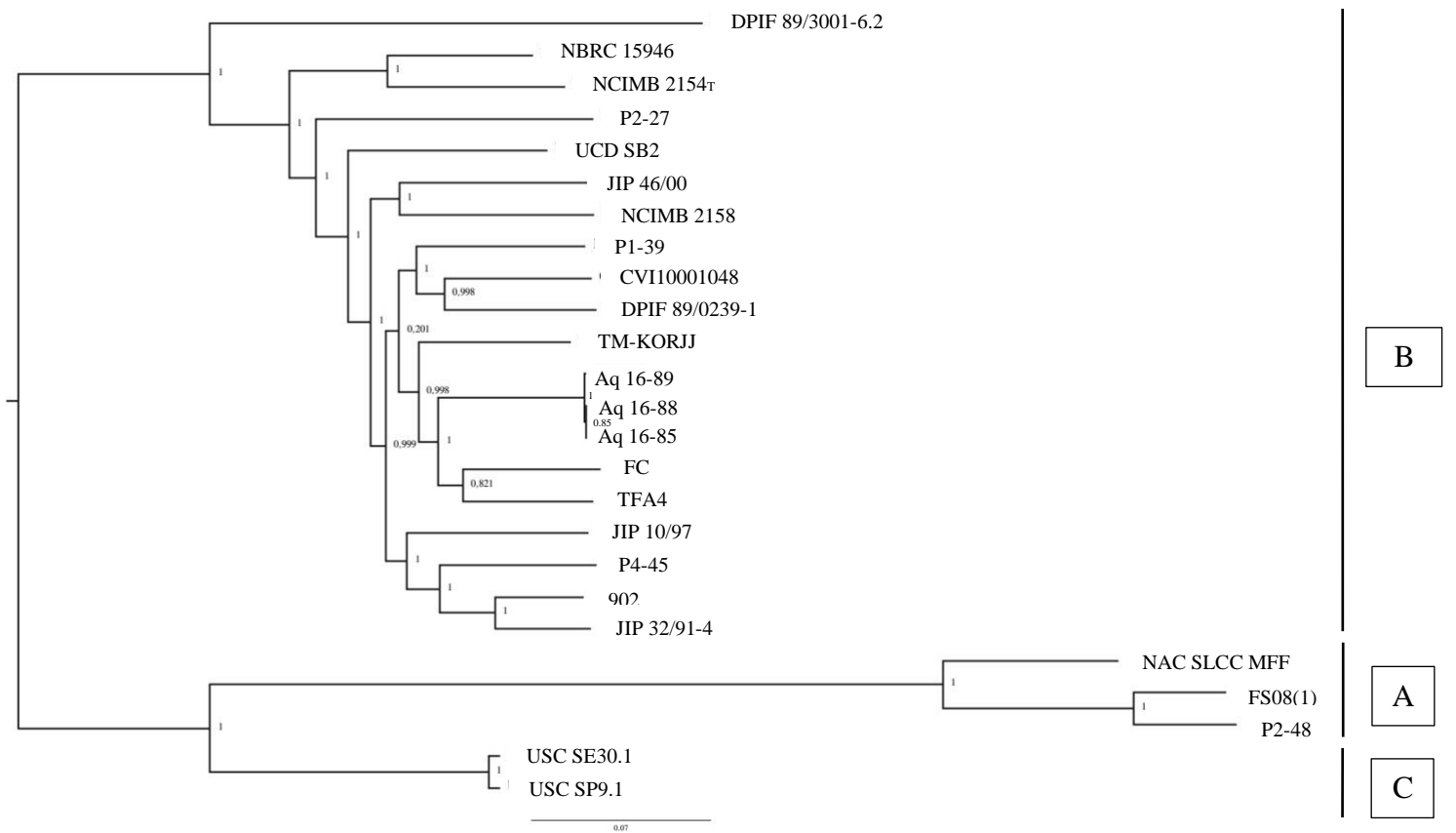

Supplement: Supplementary file 4 — Additional file 4. Maximum likelihood, Gubbins-based phylogenetic tree. The tree was obtained from the whole genome alignment of 25 T. maritimum strains at the fifth and final iteration of Gubbins. Statistical support of nodes is indicated. The three clades designated A, B, and C are labeled and delineated by vertical bars. [file 13567_2020_782_MOESM4_ESM.pdf]

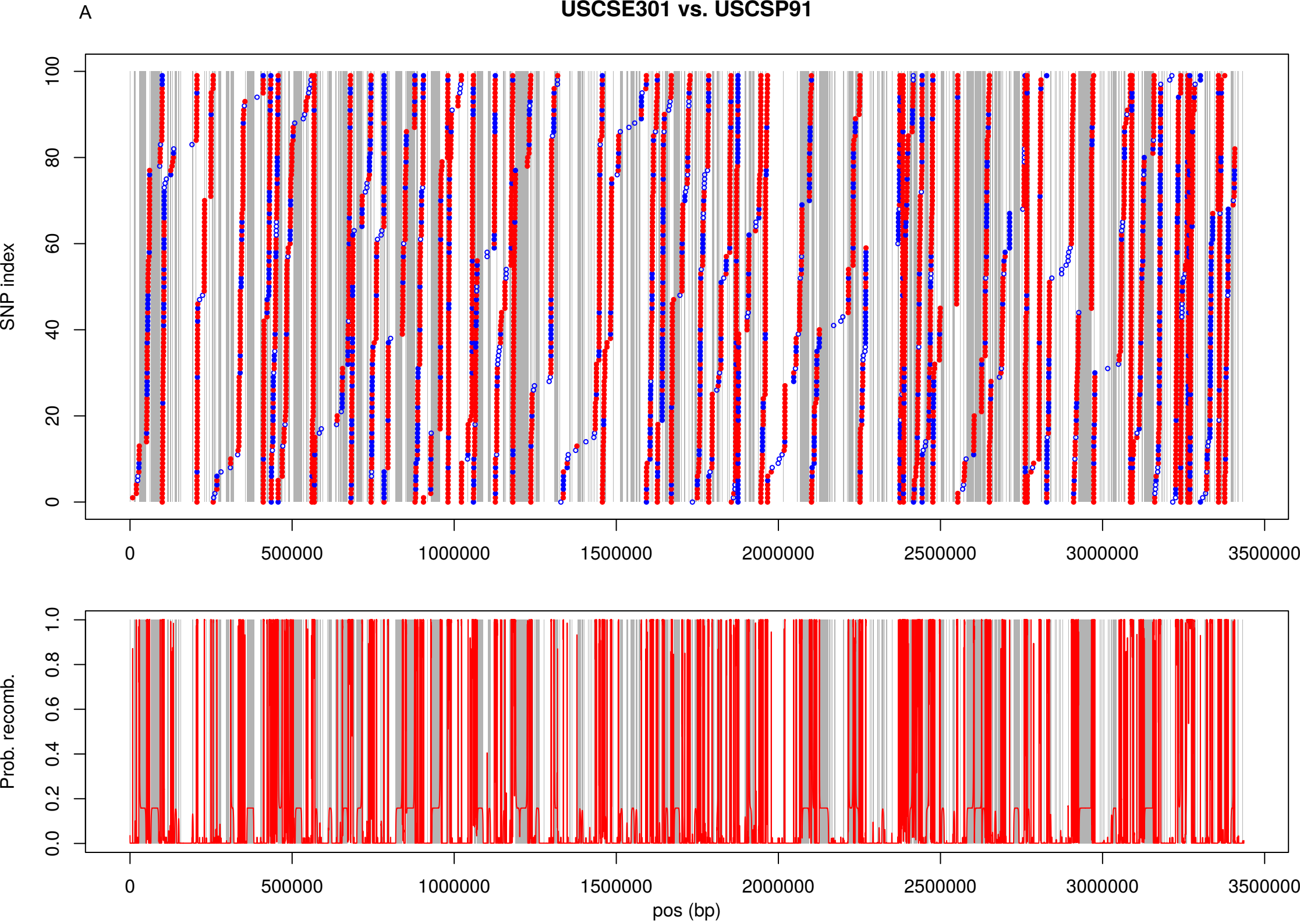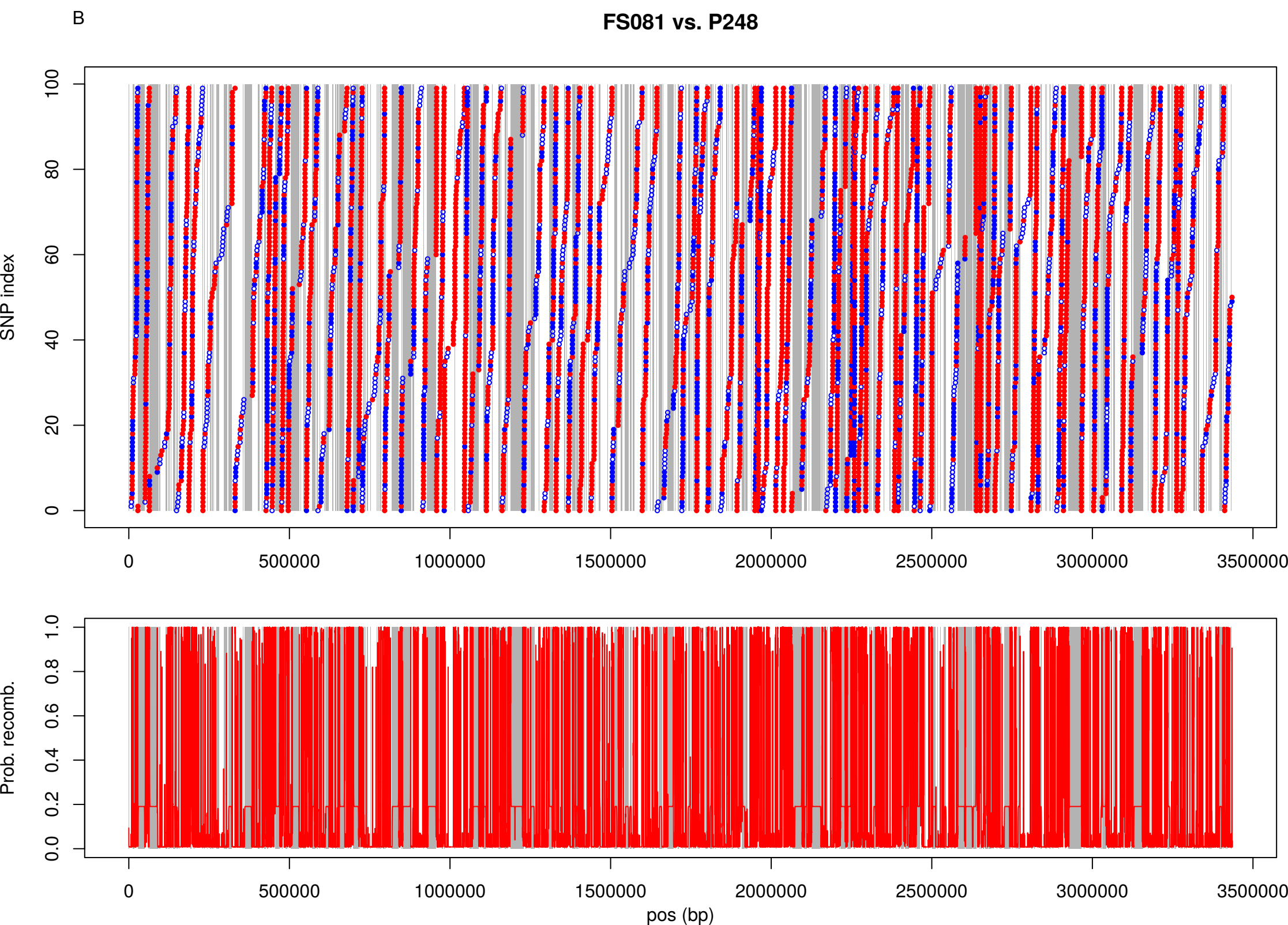

Supplement: Supplementary file 5 — Additional file 5. Overview of recombination tracts between two pairs ofT. maritimumisolates. SNPs and recombination tracts between two closely related isolates. In 5A the strains compared are USC SP9.1 and USC SE30.1. In 5B the strains compared are FS08(1) and P2−48. (Upper) Positions of the SNPs along the genomes. SNP index is reset every 100 SNPs for this representation. Each dot corresponds to one SNP in the comparison between the two considered isolates. Colors distinguish two types of polymorphism: in blue, polymorphism observed only between the two considered genomes; in red, polymorphism also observed among the other sequenced genomes. Areas in gray correspond to regions not covered by our alignments. SNPs in regions where probability is < 0.5 (i.e. outside predicted recombination tracts) are represented by open symbols (blue circles). (Lower) Probability of recombination tract as computed with the HMM. Estimation of the % of genome in recombination tracts is 15.8 and 19.1 for (A) and (B), respectively. Estimation of the average length of recombination tracts is 885 bp and 328 bp. for (A) and (B), respectively. Estimation of the average nucleotide diversity inside recombination tracts is 0.013/bp and 0.014/bp for (A) and (B), respectively. Estimation of the average nucleotide diversity outside recombination tracts is 9.9e-5/bp and 3.5e-4/bp for (A) and (B), respectively. Estimation of the number of SNPs inside recombination tracts is 5266 and 7013 for (A) and (B), respectively. Estimation of the number of SNPs outside recombination tracts is 216 and 737 for (A) and (B), respectively. Estimation of the number of SNPs due to mutations (extrapolated from non − recombined regions) is 256 and 910 for (A) and (B), respectively. Estimation of the ratio r/m is 20.6 and 7.7 for (A) and (B), respectively. [file 13567_2020_782_MOESM5_ESM.pdf]

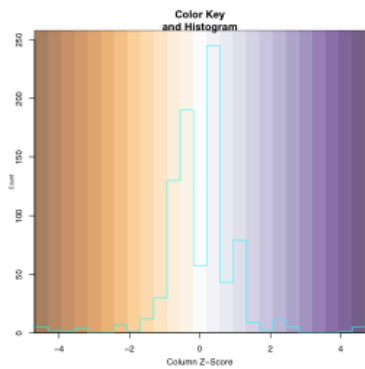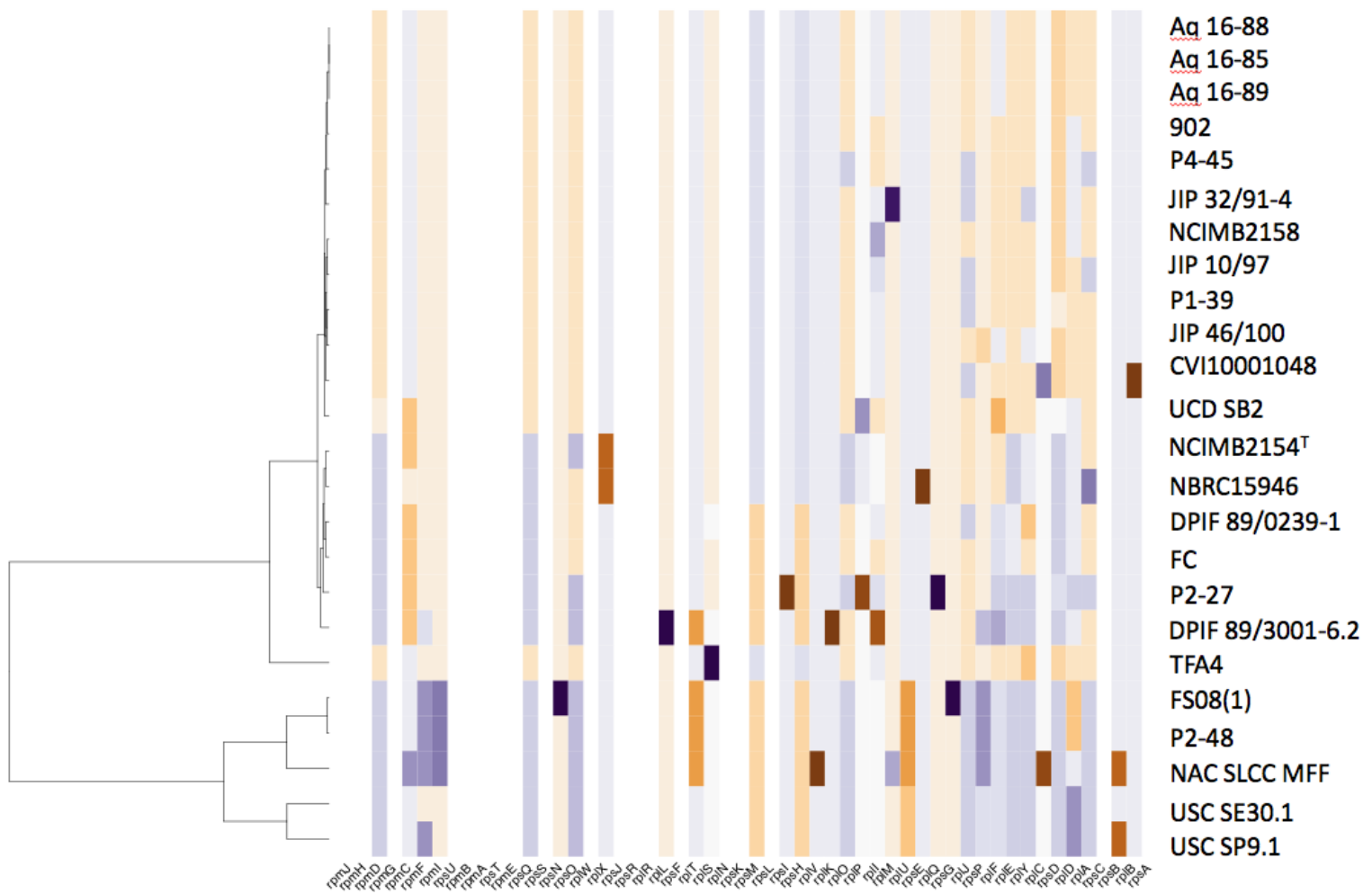

Supplement: Supplementary file 6 — Additional file 6. Heatmap displaying the diversity of ribosomal protein weights. Lines correspond to strains and columns correspond to ribosomal proteins in ascending order by weight (from left to right). White lines indicate no variation of the weight of the corresponding proteins (i.e., monomorphic proteins). In purple, proteins with weight higher than the mean and in orange proteins with weight lower than the mean (i.e., polymorphic proteins). [file 13567_2020_782_MOESM6_ESM.pdf]

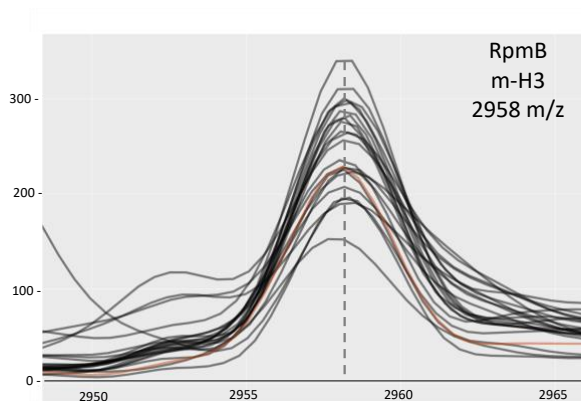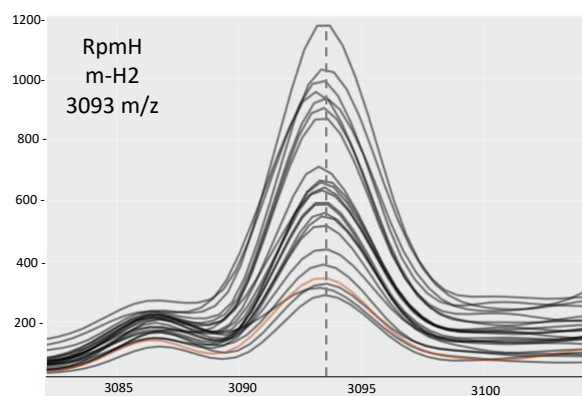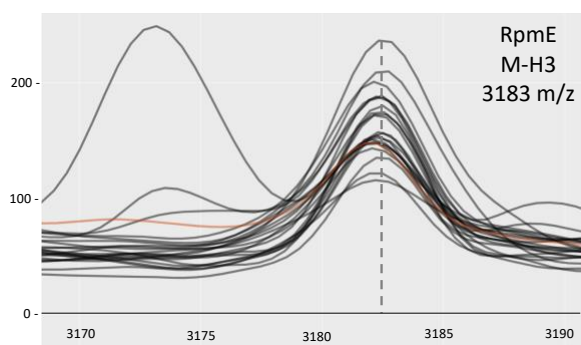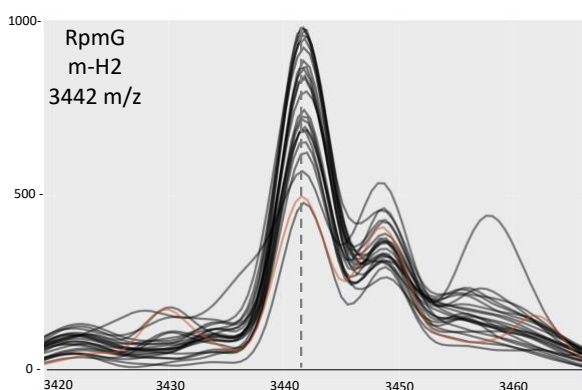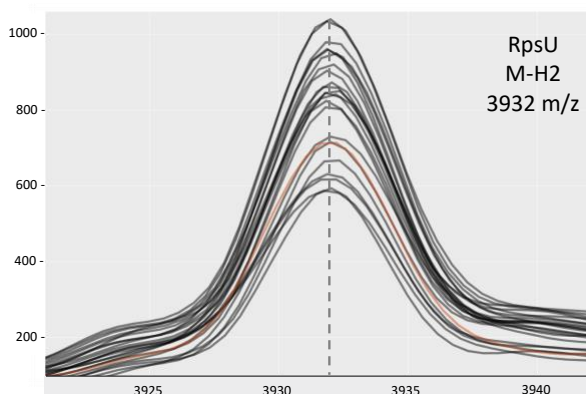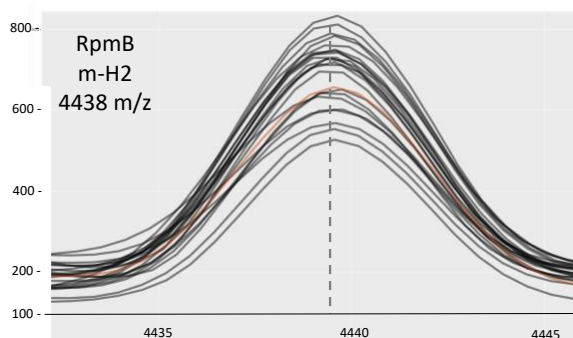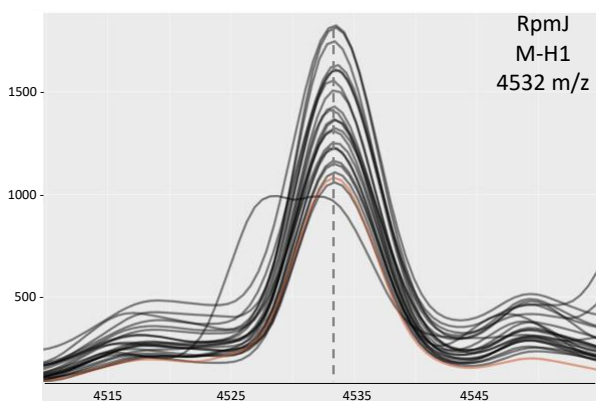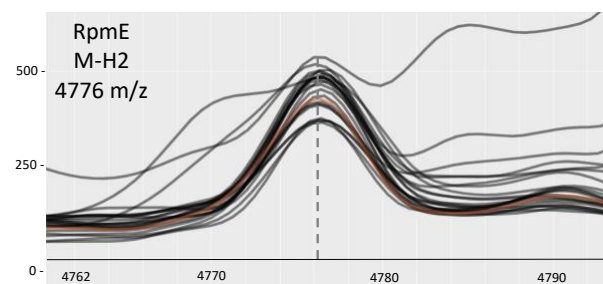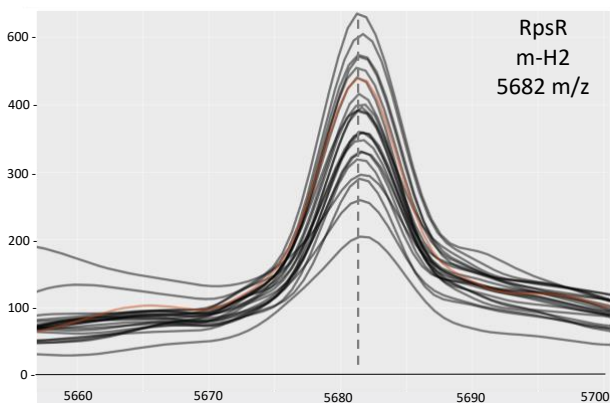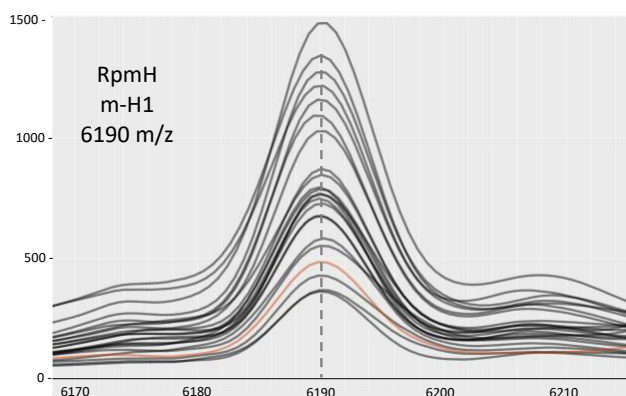

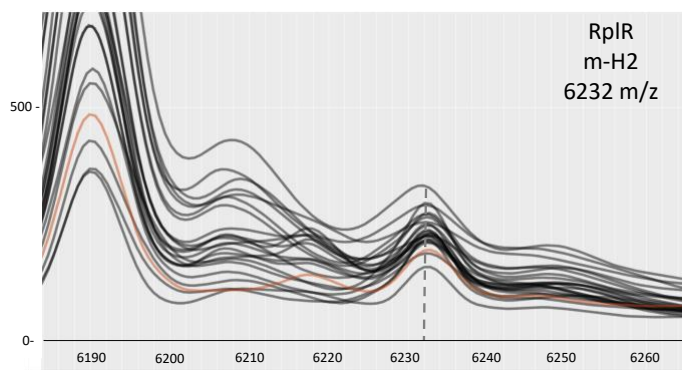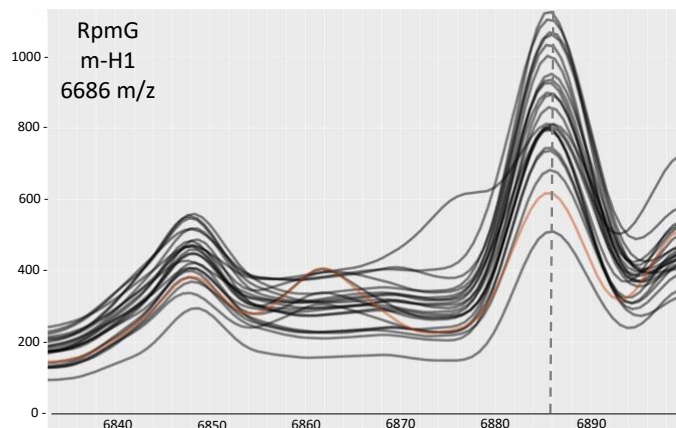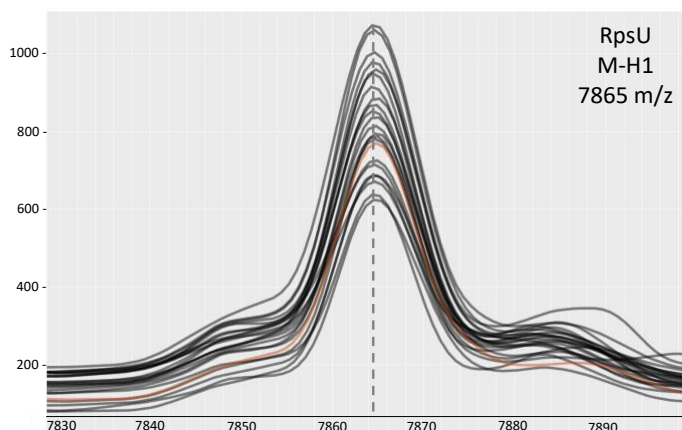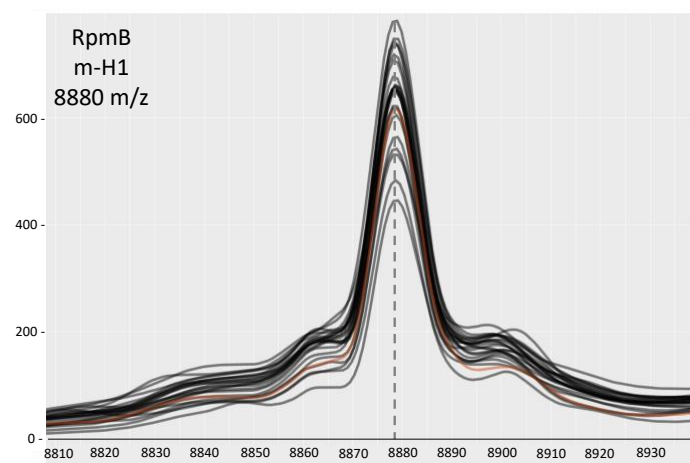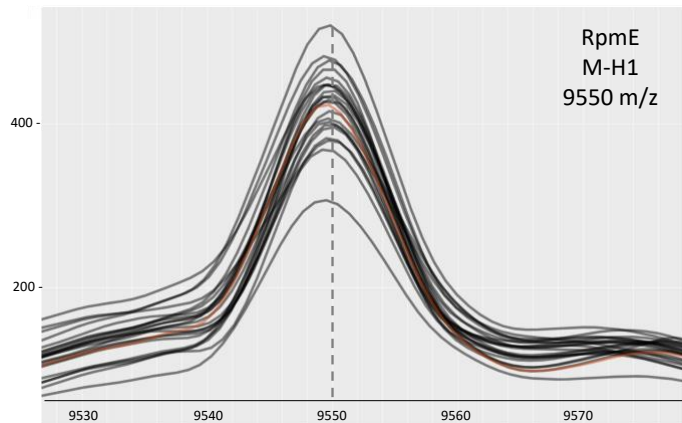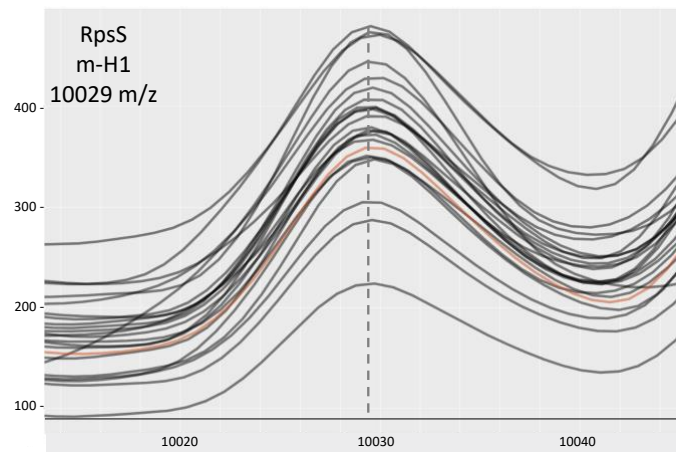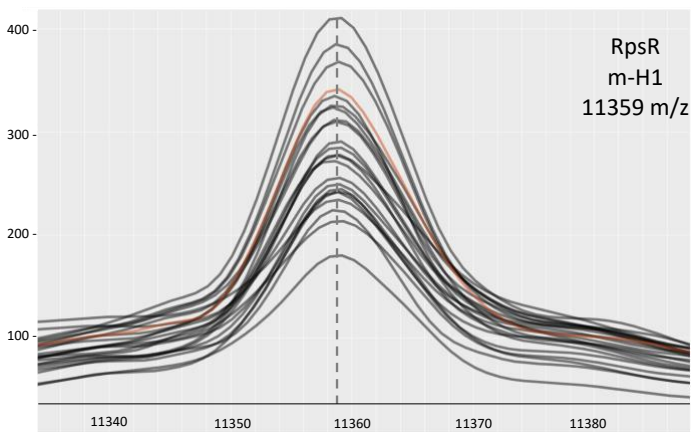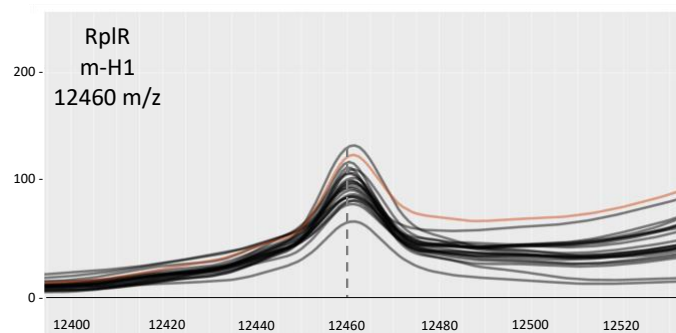

Supplement: Supplementary file 7 — Additional file 7. Monomorphic biomarker peaks. Screenshots of the 18 conserved peaks produced by 9 ribosomal monomorphic proteins with several degrees of ionization. The m/z values are highlighted by dotted lines and cover the entire spectrum. The red curve corresponds to the T. maritimum type strain average spectra. For each peak, the corresponding ribosomal protein is indicated with the degree of ionization (H1, H2 and H3 corresponding to 1, 2 and 3 H+) and the presence (M) or absence (m) of the first methionine. Color code: red line for the T. maritimum type strain NCIMB 2154T and black for the sequenced T. maritimum isolates. [file 13567_2020_782_MOESM7_ESM.pdf]

**(A)**

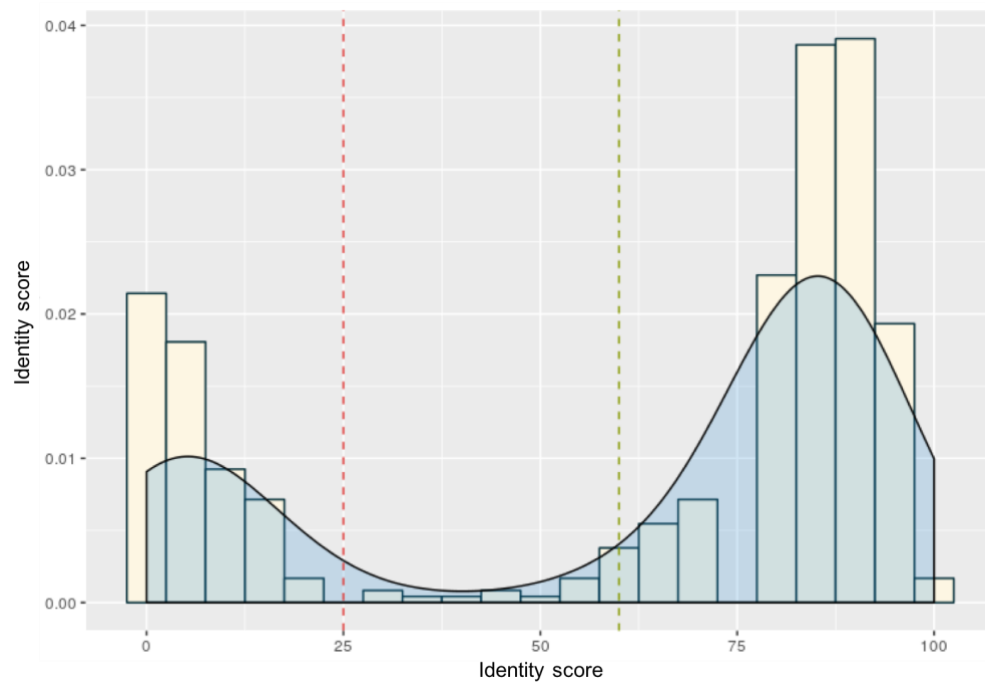

**(B)**

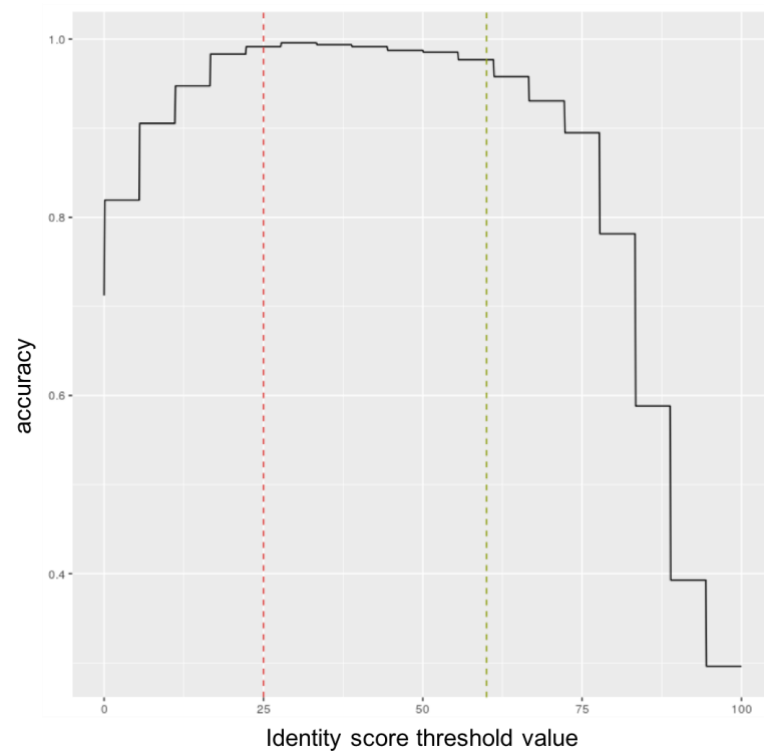

Supplement: Supplementary file 8 — Additional file 8. Quality control andT. maritimumspecies identification. The full dataset is composed of representatives of 24 Tenacibaculum species including 135 isolates belonging to the species T. maritimum. It encompasses 476 independent acquisitions (one acquisition corresponds to an average spectrum of several technical replicates) corresponding to 5102 spectra including technical and biological replicates. This dataset was divided into two groups: the positive control group (T. maritimum isolates) and the negative control group (the type strains of 23 other Tenacibaculum species). In order to confirm that an isolate belongs to the species T. maritimum, the spectra were scanned to identify the 18 T. maritimum monomorphic biomarkers. However, some of these biomarkers could be absent from a number of T. maritimum strains. Reciprocally, strains that do not belong to the T. maritimum species may possess some T. maritimum monomorphic biomarkers. In order to set up a T. maritimum species identity threshold, a value corresponding to the number of monomorphic biomarkers identified in a single sample was computed. The monomorphic biomarkers frequency plot obtained shows a bimodal distribution (Figure A). All samples with a score above 60% correspond to bona fide T. maritimum isolates while all samples with a score below 25% belong to other Tenacibaculum species. True and false positives correspond to isolates correctly and incorrectly identified as T. maritimum (TP and FP), respectively. On the other hand, true and false negatives correspond to correctly and incorrectly rejected isolates (TN and FN, respectively). Positive isolates correspond to those having an identity value above a defined threshold. Using the full dataset, the number of TP and FP and the number of TN and FN were counted. The accuracy of the tool [i.e., (TP + TN)/(TP + TN + FP + FN)] was then computed by increasing the threshold value from 0% to 100% by a 0.1% step. One could observe than the accura [file 13567_2020_782_MOESM8_ESM.pdf]
