# Supplementary material for: Genetic diversity and population structure of Tenacibaculum maritimum, a serious bacterial pathogen of marine fish: from genome comparisons to high throughput MALDI-TOF typing
Source: Vet Res. 2020 May 7;51:60. doi: 10.1186/s13567-020-00782-0 (PMC7204230; doi:10.1186/s13567-020-00782-0)
Supplement: Supplementary file 9 — Additional file 9. Amino-acid polymorphism of the 9 retained biomarkers. Protein sequence alignments of the 9 ribosomal proteins selected as polymorphic biomarkers and their corresponding isoform (IF). [file 13567_2020_782_MOESM9_ESM.pdf]

```
RpmD IF1   MSKIKITQVRSQIGRFKNQKRTLEALGLRKMNQTVEHEATPSIVGMVNTVKHLISVEEVK   60
RpmD IF2   MSKIKVTQVRSQIGRLKSQKRTLEALGLRKINQTVEHDATSTILGMVNKVQHLVSVEEIK   60
RpmD IF3   MSKIKVTQVRSQIGRLKSQKRTLEALGLRKINQTVEHDATATILGMVNKVQHLVSVEEIK   60
```

```
RpmC I[illegible]   MKQSEIKELSIADLQEQLVALKKNYTDLKMAHAITPLENPLQLKSLRRSVARIATELTKRELQ   63
RpmC I[illegible]   MKQSEIKELSIADLQEQLVALKKNYTDLKMAHAITPLENPLQLRSLRRSVARIATELTKRELQ   63
RpmC IF3   MKQSEIKELSIADLQEQLVALKKKYTDLKMAHAITPLENPLQLKSLRRSVARIATELTKRELQ   63
RpmC IF4   MKQSEIKELSIADLQEQLVALKKNYTDLKMAHAITPLENPLQLRSLRRSVARIVTELTKRELQ   63
```

```
RpsP IF1   MPVKIRLQRHGKKGKPFYWVVAADSRAKRDGRFLEKIGTYNPNTNPATIELDVDSAVKWLQNGAQPTDTARALLSYKGALLKNHLAGGVRKGALTEEQAAAKFEAWLEEKEGKVS   115
RpsP IF2   MPVKIRLQRHGKKGKPFYWVVAADSRAKRDGRFLEKIGTYNPNTNPATIELDVDSAVKWLQNGAQPTDTARALLSYKGALLKNHLAGGVRKGALTEEQAAAKFEAWLEEKEGKVS   115
RpsP IF3   MPVKIRLQRHGKKGKPFYWVVAADSRAKRDGRFLEKIGTYNPNTNPATIELDVDSAVKWLQNGAQPTDTARALLSYKGALLKNHLAGGVRKGALTEEQAAAKFEAWLEEKEGKVS   115
RpsP IF4   MPVKIRLQRHGKKGKPFYWVVAADSRAKRDGRFLEKIGTYNPNTNPATVELDVDSAVKWLQNGAQPTDTARTLLSYKGALLKNHLVGGVRKGALTEEQAAAKFEAWLEEKEGKVS   115
RpsP IF5   MPVKIRLQRHGKKGKPFYWVVAADSRAKRDGRFLEKIGTYNPNTNPATIELDVDSAVKWLQNGAQPTDTARALLSYKGALLKNHLAGGVRKGALTEEQAAAKFEAWLEEKEGKVS   115

RpsP IF1   TKEADLAKAKEAAKAKALEAEKAVNEARIAAAVPAVEEESEATTEEAPEAAAKSEE   171
RpsP IF2   TKETDLAKAKEVAKAKALEAEKAVNEARIAAAVPAVEEESEATTEEAPEAAAKSEE   171
RpsP IF3   TKETDLAKAKEAAKAKALEAEKAVNEARIAAAVPAVEEESEATTEEAPEAAAKSEE   171
RpsP IF4   TKEADLAKAKEAAKAKALEAEKAVNEARIAAAVPAVEEESEATTEEAPEAAAKSEE   171
RpsP IF5   TKEADLAKAKEAAKAKALEAEKAVNEARVAAAVPAVEEESEATTEEAPEAAAKSEE   171
```

```
RpsT IF1   MANHKSALKRIRSNEAKRLRNKYQHKTTRNAVRKLRATEDRKEAEGMFSKVVSMLDKLAKNNIIHKNKASNLKSKLAKHVAAL   83
RpsT IF2   MANHKSALKRIRSNEAKRLRNKYQHKTTRNAVRKLRATEDKKEAEGMFSKVVSMLDKLAKNNIIHKNKASNLKSKLAKHVAAL   83
```

```
RpsN IF1   AKESMKARERKRAKTVAKFAEKRKALKEAGDYEALQKLPKNASPIRMHNRCKLTGRPKGYMRQFGISRVTFREMANQGLIPGVTKASW   89
RpsN IF2   AKESMKARERKRAKIVAKFAEKRKALKEAGDYEALQKLPKNASPIRMHNRCKLTGRPKGYMRQFGISRVTFREMANQGLIPGVTKASW   89
```

```
RpsO IF1   MYLTKEVKEGIFEKHGKGKNDTGTSEGQIALFTFRINHLTEHLKKNRKDFNTERSLVKMVGKRRSLLDYLKKKDINRYRAIIKELGIRK   89
RpsO IF2   MYLTKEVKEGIFEKHGKGKNDTGTSEGQIALFTFRINHLTEHLKNNRKDFNTERSLVKMVGKRRSLLDYLKKKDINRYRAIIKELGIRK   89
```

```
RpsQ IF1   MEKRNLRKERIGVVSSNKMEKSIVVNEVKRVKHPMYGKFVLKTKKYVAHDEKNDCNIGDTVRIMETRPLSKSKRWRLVEILERAK   85
RpsQ IF2   MEKRNLRKERIGVVSSNKMEKSIVVSEVKRVKHPMYGKFVLKTKKYVAHDEKNDCNIGDTVRIMETRPLSKSKRWRLVEILERAK   85
```

```
RplX IF1   MQKFKIKSGDTVKVIAGDHKGSEGKVLRILKEKNRVVVEGVNMISKHTKPSAANPQGGIVKKEAPIHVSNLALVENGEAVRVGYRIEGDKKVRFSKKSDKAI   102
RplX IF2   MQKFKIKSGDTVKVIAGDHKGSEGKVLRILKEKNRVVVEGVNMISKHTKPSAANPQGGIVKKEAPIHVSNLALVENGEAVRVGYRMEGDKKVRFSKKSDKAI   102
```

```
RplT IF1   MPRSVNSVASRRRRKKILKQAKGYFGRRKNVYTVAKNAVEKAMTYAYRDRKNNKRNFRSLWIQRINAGARQFGMSYSQFMGKVKANDIELNRKVLADLAMNNPEAFKAIVDKIK   114
RplT IF2   MPRSVNSVASRRRRKKILKQAKGYFGRRKNVYTVAKNAVEKAMSYAYRDRKNNKRNFRSLWIQRINAGARQFGMSYSQFMGKVKANDIELNRKVLADLAMNNPEAFKAIVDKIK   114
RplT IF3   MPRSVNSVASRKRRKKILKQAKGYFGRRKNVYTVAKNAVEKAMTYAYRDRKNNKRNFRSLWIQRINAGARQFGMSYSQFMGKVKANDIELNRKVLADLAMNNPEAFKAIVDKIK   114
```
