# Supplementary material for: Genetic diversity and population structure of Tenacibaculum maritimum, a serious bacterial pathogen of marine fish: from genome comparisons to high throughput MALDI-TOF typing
Source: Vet Res. 2020 May 7;51:60. doi: 10.1186/s13567-020-00782-0 (PMC7204230; doi:10.1186/s13567-020-00782-0)
Supplement: Supplementary file 4 [file 13567_2020_782_MOESM2_ESM.docx]

	NAC SLCC MFF	P1-39	P2-27	UCD SB2	JIP 10/97	Aq 16-85	JIP 46/00	TFA4	CVI10001048	Aq 16-89	FC	P4-45	902	NCIMB 2158	Aq 16-88	NBRC 15946	NCIMB 2154T	FS08(1)	DPIF 89/3001-6.2	P2-48	JIP 32/91-4	USC SP9.1	USC SE30.1	DPIF 89/0239-1	TM-KORJJ
NAC SLCC MFF	100,00	98,41	98,45	98,38	98,38	98,37	98,36	98,37	98,37	98,37	98,39	98,40	98,38	98,38	98,37	98,49	98,41	99,26	98,31	99,28	98,38	98,67	98,65	98,38	98,38
P1-39	98,41	100,00	99,31	99,48	99,53	99,54	99,51	99,51	99,57	99,54	99,53	99,53	99,55	99,48	99,54	99,40	99,29	98,29	98,94	98,32	99,54	98,86	98,86	99,50	99,53
P2-27	98,45	99,31	100,00	99,27	99,24	99,31	99,24	99,23	99,27	99,31	99,30	99,28	99,23	99,27	99,31	99,27	99,14	98,29	98,80	98,33	99,26	98,76	98,73	99,25	99,28
UCD SB2	98,38	99,48	99,27	100,00	99,44	99,46	99,43	99,46	99,44	99,46	99,46	99,47	99,44	99,43	99,46	99,36	99,28	98,21	98,94	98,27	99,40	98,78	98,79	99,44	99,47
JIP 10/97	98,38	99,53	99,24	99,44	100,00	99,48	99,43	99,48	99,47	99,48	99,46	99,49	99,47	99,45	99,49	99,36	99,22	98,23	98,91	98,24	99,45	98,82	98,81	99,46	99,47
Aq 16-85	98,37	99,54	99,31	99,46	99,48	100,00	99,47	99,55	99,54	99,99	99,57	99,52	99,48	99,48	100,00	99,41	99,28	98,24	98,93	98,25	99,49	98,80	98,78	99,49	99,51
JIP 46/00	98,36	99,51	99,24	99,43	99,43	99,47	100,00	99,42	99,45	99,47	99,43	99,47	99,43	99,44	99,47	99,39	99,24	98,18	98,89	98,24	99,42	98,77	98,78	99,40	99,49
TFA4	98,37	99,51	99,23	99,46	99,48	99,55	99,42	100,00	99,49	99,56	99,51	99,48	99,47	99,45	99,56	99,39	99,26	98,24	98,93	98,25	99,44	98,81	98,78	99,51	99,49
CVI10001048	98,37	99,57	99,27	99,44	99,47	99,54	99,45	99,49	100,00	99,54	99,49	99,49	99,48	99,47	99,54	99,41	99,26	98,21	98,93	98,23	99,46	98,82	98,80	99,50	99,50
Aq 16-89	98,37	99,54	99,31	99,46	99,48	99,99	99,47	99,56	99,54	100,00	99,56	99,52	99,48	99,48	99,99	99,41	99,28	98,25	98,94	98,26	99,50	98,80	98,78	99,49	99,52
FC	98,39	99,53	99,30	99,46	99,46	99,57	99,43	99,51	99,49	99,56	100,00	99,47	99,49	99,43	99,55	99,41	99,28	98,22	98,92	98,29	99,43	98,82	98,81	99,46	99,50
P4-45	98,40	99,53	99,28	99,47	99,49	99,52	99,47	99,48	99,49	99,52	99,47	100,00	99,57	99,46	99,51	99,38	99,25	98,25	98,90	98,25	99,53	98,81	98,82	99,45	99,52
902	98,38	99,55	99,23	99,44	99,47	99,48	99,43	99,47	99,48	99,48	99,49	99,57	100,00	99,42	99,47	99,38	99,24	98,21	98,93	98,25	99,63	98,82	98,79	99,45	99,50
NCIMB 2158	98,38	99,48	99,27	99,43	99,45	99,48	99,44	99,45	99,47	99,48	99,43	99,46	99,42	100,00	99,49	99,39	99,27	98,18	98,90	98,23	99,45	98,77	98,76	99,42	99,47
Aq 16-88	98,37	99,54	99,31	99,46	99,49	100,00	99,47	99,56	99,54	99,99	99,55	99,51	99,47	99,49	100,00	99,40	99,28	98,24	98,92	98,26	99,49	98,80	98,78	99,49	99,50
NBRC 15946	98,49	99,40	99,27	99,36	99,36	99,41	99,39	99,39	99,41	99,41	99,41	99,38	99,38	99,39	99,40	100,00	99,55	98,35	98,96	98,37	99,36	98,77	98,78	99,38	99,39
NCIMB 2154T	98,41	99,29	99,14	99,28	99,22	99,28	99,24	99,26	99,26	99,28	99,28	99,25	99,24	99,27	99,28	99,55	100,00	98,29	98,86	98,31	99,24	98,72	98,71	99,23	99,27
FS08(1)	99,26	98,29	98,29	98,21	98,23	98,24	98,18	98,24	98,21	98,25	98,22	98,25	98,21	98,18	98,24	98,35	98,29	100,00	98,19	99,54	98,24	98,66	98,65	98,23	98,21
DPIF 89/3001-6.2	98,31	98,94	98,80	98,94	98,91	98,93	98,89	98,93	98,93	98,94	98,92	98,90	98,93	98,90	98,92	98,96	98,86	98,19	100,00	98,21	98,91	98,65	98,69	98,95	98,92
P2-48	99,28	98,32	98,33	98,27	98,24	98,25	98,24	98,25	98,23	98,26	98,29	98,25	98,25	98,23	98,26	98,37	98,31	99,54	98,21	100,00	98,25	98,65	98,64	98,26	98,24
JIP 32/91-4	98,38	99,54	99,26	99,40	99,45	99,49	99,42	99,44	99,46	99,50	99,43	99,53	99,63	99,45	99,49	99,36	99,24	98,24	98,91	98,25	100,00	98,74	98,73	99,42	99,46
USC SP9.1	98,67	98,86	98,76	98,78	98,82	98,80	98,77	98,81	98,82	98,80	98,82	98,81	98,82	98,77	98,80	98,77	98,72	98,66	98,65	98,65	98,74	100,00	99,67	98,80	98,78
USC SE30.1	98,65	98,86	98,73	98,79	98,81	98,78	98,78	98,78	98,80	98,78	98,81	98,82	98,79	98,76	98,78	98,78	98,71	98,65	98,69	98,64	98,73	99,67	100,00	98,82	98,76
DPIF 89/0239-1	98,38	99,50	99,25	99,44	99,46	99,49	99,40	99,51	99,50	99,49	99,46	99,45	99,45	99,42	99,49	99,38	99,23	98,23	98,95	98,26	99,42	98,80	98,82	100,00	99,48
TM-KORJJ	98,38	99,53	99,28	99,47	99,47	99,51	99,49	99,49	99,50	99,52	99,50	99,52	99,50	99,47	99,50	99,39	99,27	98,21	98,92	98,24	99,46	98,78	98,76	99,48	100,00
